# Different Methods of Winning, Losing, and Training in Combat Sports and Their Relationship with Overall Competitive Winningness

**DOI:** 10.1155/2024/5531981

**Published:** 2024-02-21

**Authors:** Oliver R. Barley, Craig A. Harms

**Affiliations:** ^1^School of Medical and Health Sciences, Centre for Human Performance, Edith Cowan University, Joondalup, WA, Australia; ^2^School of Arts and Humanities, Psychology and Criminology, Edith Cowan University, Joondalup, WA, Australia

## Abstract

This study aimed to investigate how overall competitive winningness in combat sports depended on patterns of victory and loss, as well as training habits. Competitors (*N* = 280) from several combat sports participated in the study. The online survey included questions on self-reported patterns of victory (and loss), training habits, general demographics (e.g., age), and sport-specific information (e.g., stage of career and competitive style). Overall, it was found across four models that reflected diversity of winningness in combat sports that the most important predictors of competitive winningness were loss by points (negative), loss by submission (negative), loss (negative) or victory (positive) by throw or technical fall, and loss (negative) or victory (positive) by knockout. The findings applied to amateur and regional/state athletes, and rarely to karate or tae kwon do. Findings around demographics or training habits were largely unremarkable, outside of a relationship between higher training loads and less career winning in wrestlers. Results show that while winning via a finishing sequence (e.g., knockout or submission) is preferable to the judge's decision or points, the matter of victory is less important than the methods by which an athlete loses. In grappling-only sports, we observed a trend that more losses via finishing sequence were worse for careers than losing by points. In fact, having most of one's losses coming via judge's decision or points was beneficial in wrestling and judo, perhaps due to athletes taking less risks and having better defence. These findings may aid practitioners developing effective tactics and training programs.

## 1. Introduction

Combat sports include a range of sports involving one-on-one fighting under a specific ruleset. However, combat sports vary between each other. Sports can include striking with the limbs (e.g., punches, kicks, knees, or elbows), or grappling actions (e.g., takedowns, joint manipulation, and chokes) [1]. A wide range of demographics currently compete in combat sports across a wide range of competitive levels extending from amateur and/or regional, to professional and/or international [1]. Unlike many team sports, while the full bout time can be completed and total points or a subjective judge's decision used to determine the winner, combat sports bouts can be won or lost prior to the completion of allocated match time. Bouts can conclude when a certain number of points are accrued or there is a significant points differential (e.g., the 20 pt advantage rule in tae kwon do), an opponent is knocked unconscious or is deemed unable to continue by an official, a specific technique is executed (e.g., a pin in wrestling), or an athlete submits due to grappling attacks [2]. The ability to finish bouts within the allocated bout time creates an interesting dynamic that has physical, physiological, and tactical implications.

Determining the factors that influence overall winningness in combat sports is complicated due to their highly physical and tactical nature. The current body of research has typically approached this area in one of two ways. First, investigating physical and physiological profiles in varying combat sports and typically found that more winning athletes are stronger and fitter than their contemporaries [3–7]. Second, previous research has reviewed footage of competitions to identify strategies related to overall winningness. Such studies have been completed in MMA [8], boxing [9], judo [10], and wrestling [11]. For example, research by James et al. (2017) observed that landing strikes on the ground and completing takedowns was a successful strategy to winning bouts in MMA. While such methods provide important information on overall winningness in combat sports, there are certain areas that remain relatively unexplored.

There are a range of factors that could potentially influence winning in combat sports in combat sports such as competitive patterns (specific methods of victory and loss) and training habits (training volume and type of training) [1]. It is unclear how differing competitive patterns influence overall competitive winningness in combat sports. It is plausible that general trends of winning and losing are related to overall competitive performance. For example, it is unclear if athletes who acquire most of their wins via points will have a more winning record than those athletes who acquire most of their wins via other methods such as submissions or pin.

Previous research has reported that physical conditioning is important for combat sports success [3–7], yet there are minimal data within combat sports investigating how different training habits may relate to competitive success (i.e., winningness). There are some data that have investigated general training habits in combat sports athletes [1, 12, 13], including how many sport specific and nonspecific sessions are completed weekly. However, this research has not examined such habits relative to overall competitive winningness, despite the obvious importance of how training is conducted in competitive athletes.

The purpose of this study was to examine how overall competitive winningness in combat sports depended on patterns of victory and loss as well as habits of combat and noncombat training that athletes engage in. We controlled personal demographic measures such as age and body mass index (BMI), as such factors may influence experience in a sport, as well as their viability for fighting at different weight classes [14, 15]. We also took into account sport-specific demographic factors such as career stage and competitive style in the models because experience and aggression are plausible factors to influence the competitive record [16, 17]. Given there are several different combat sports with many different competitive levels (e.g., amateurs, regional, and elite), this study will aim to examine how such factors outlined impact on competitive performance across different combat sports, and competitive levels. Examining overall trends of athlete's competitive careers and determining relationships with more winning records may provide valuable insights into the best training and competing strategies for winning across combat sports.

## 2. Materials and Methods

### 2.1. Procedures

The study's procedures were approved by our institution's Human Ethics Committee (Research ethics identification: 2019-00278-Barley). With permission, several combat sport organisations, gyms, and message boards from around the world promoted the survey. Participants followed a link to the survey after being emailed it or accessing it directly from promotional material. Participants competed the survey online using Qualtrics (Qualtrics, August to October 2019, Qualtrics, Provo, Utah, USA). Prior to data collection, participants indicated their consent by accepting the terms and conditions on the first page of the survey.

Participants completed a section focused on questions that were demographic and general in nature, such as country of residence as well as age and biological sex. Additionally, questions on some physical attributes, such as height and weight (to calculate BMI), were included. Participants were allowed to enter the results in a range of units of measurements which were later converted to the metric system. Four demographic questions were sport-specific in nature. Participants were asked to rate their competitive style (VAS: 0 = “defensive”; 100 = “aggressive”); stage of career (VAS: 0 = “start of career”; 50 = “middle of career”; 100 = “end of career”); primary combat sport in the previous twelve months; and highest level of competition (amateur, regional/state, or elite). These questions were adapted from previous research [18, 19]. Participants answered questions about current training habits such as typical frequency of combat and noncombat training (e.g., strength training) each week without and with a competition in the near term.

Participants were questioned about their competitive record as well as typical methods of victory or loss. Participants reported competitive results over their career including estimated number of victories, losses, and number of competitions, so that at winning (i.e., ratio of victories to competitions, expressed as a percentage) and losing (i.e., ratio of victories to competitions, expressed as a percentage), a record could be calculated. Participants also reported, on a five-point Likert scale (1 = none; 5 = all), their typical frequency for methods of victory or loss (as appropriate to their primary sport): points or judge's decision; knockout, technical knockout, or corner/doctor's stoppage; submission or pin; disqualification; or Ippon, Wazari-ari, or technical fall.

### 2.2. Subjects

A total of 298 combat sport athletes aged 18 years and over actively involved in combat sport associations or gyms and had a competitive record were participated in the study. As noted by Barley and Harms (2021), participants mostly residing in the USA, UK, Australia, and Canada (80.1%), were males, (*n* = 256, 85.9%), primarily participated in mixed martial arts (MMA: *n* = 30), Muay Thai/kickboxing (MT/KB: *n* = 46), boxing (*n* = 61), Brazilian jiu jitsu (BJJ: *n* = 58), wrestling (*n* = 43), judo (*n* = 26), or karate and taekwondo (K & T, *n* = 29; which, due to small numbers, combined participants); and participated in the previous 12 months at amateur (*n* = 115); and competed at regional/state (*n* = 99) or elite (national, international, semiprofessional, and professional combined): (*n* = 84) level. Participants were aged 28.42 ± 9.5 years; had a self-reported BMI of 25.32 ± 4.90; and were close to the middle of their career (*M* = 45.71 ± 32.74).

### 2.3. Statistical Analysis

Where analyses involved winning or losing record, only participants with nonzero winning or losing records were retained for analysis. After some initial screening of the data (Supplementary File A), two broad categories of winning and losing methods emerged—common and less common methods. Common methods of winning and losing included victory by points or judge's decision, loss according to points or judge's decisions, victory by knockout, technical knockout, or corner/doctors' stoppage (referred hereafter as “knockout”), and victory or loss by submission or pin. Less common methods of winning and losing included loss by knockout, victory and loss by disqualification, victory or loss by Ippon, Waza-ari, or technical fall (referred hereafter as “throws or TF”).

Initial analysis focused on examining association between the two measures of performance in competition—winning and losing record. If the correlation between winning and losing record was very large, then findings relating to winning record were reported.

The main analysis focused on regressing competition performance on a set of predictors—personal (age, gender, and BMI) as well as sport-specific demographic (career stage and competitive style) measures, current training habits (combat and noncombat; with or without a competition in the near term), and methods of victory and loss.

Given the heterogeneity of combat sports regarding methods of victory or loss, four models—based on commonality of methods of victory and loss across combat sports—were examined. In model one, which applied to all participants, the methods of victory included were points or judge's decision or by disqualification. In model two, which applied to MMA, MT/KB, boxing, and K & T, the methods of victory or loss included were points or judge's decision, disqualification, and knockout. In model three, which applied to MMA, BJJ, wrestling, and judo, the methods of victory or loss included were points or judge's decision, disqualification, and submission or pin. In model four, which applied to wrestling, and judo, the methods of victory or loss included were points or judge's decision, disqualification, and submission or pin, and throws or TF. To reduce the number of predictors in each model, preliminary analysis (which occurred in steps, as described in separate appendices for each model) was used to establish which measures were making a nonsignificant contribution to competition performance. In all models, competition performance was regressed on personal as well as sport-specific demographic measures and current training habits.

As the number of predictor variables in each model was large (e.g., 14 predictors for Model 1) such that the models were underpowered with only a sample of 298 (and only 280 had a competitive record), it was decided to conduct an initial standard regression analysis to identify predictors that made a statistically significant contribution to competitive performance. Multigroup analysis—by level of competition and by primary sport—was conducted for all bivariate associations to identify any variables to be retained for the final analysis. Once the final set or predictors was determined, a final regression analysis was conducted, with multigroup analysis also conducted for each model to examine effects according to competition level as well as the competitors primary combat sport.

Descriptive data and regression analyses were conducted using SPSS (version 25). Alpha (*α*) was set at 0.05 for all tests because the overall findings were exploratory in nature. Cohen's criterion [20] was used to evaluate effect size for all parametric analyses such that *d* < 0.20 indicates a small effect, *d* = 0.21–0.50 indicates a medium effect, and *d* > 0.80 indicates a large effect. Values for *d* are reported in the tables, with description of those effects reported in text. Multigroup analyses were conducted in Mplus Version 8.3 [21].

## 3. Results

### 3.1. Association between Winning and Losing Record

For the participants who had a nonzero competitive record (*n* = 246), the correlation between winning record (63.07% ± 19.61) and losing record (34.44% ± 18.79) was −0.94 (*p* < 0.01). No moderation effects for level of competition, *χ*^2^ = 5.13, df = 2, *p*=0.08, or primary sport, *χ*^2^ = 6.77, df = 6, *p*=0.34, were observed. Based on these results, the findings for winning record only were reported.

**Model 1 .**

Winning record based on victory and loss by points as well as disqualification—for all sports

After preliminary analysis for this model (Supplementary File B), winning record was regressed on competitive style, frequency of *noncombat* training sessions each week *without* a competition in the near term, frequency of *noncombat* training *with* a competition in the near term, and loss by points or judge's decision (Table 1). Findings for frequency of *noncombat* training sessions each week *with* a competition in the near-term were not reported in this table due to a lack of impact for explaining variance in winning record for this model (Supplementary File B).

The overall model explained a statistically significant but small amount variance in winning record. Findings from the moderation analyses indicated that the model explained a statistically significant amount of variance in winning record for regional/state (moderate) and amateur (large) athletes only for boxing, BJJ, and wrestling (moderate), as well as MMA (large).

Two broad unique effects were observed for this model. Athletes with a better winning record reported a more aggressive style (small effect), with this effect being limited to amateur and regional/state athletes as well as for BJJ athletes (small effects).

Athletes who reported a better winning record also reported fewer losses by points, with this effect specifically limited to amateurs and MT/KB and boxing (moderate to large effects). In contrast, athletes who reported a better winning record in wrestling reported more losses by points (moderate effect).

One specific unique effect was observed for this model. BJJ athletes who had a better winning record also reported a greater frequency of noncombat training sessions each week without a competition in the near term (large effect).

**Model 2 .**

Winning record based on victory and loss by points, disqualification and knock-out—for MMA, MT/KB, Boxing, and K & T

After the preliminary analysis for this model (see Supplementary File C), winning record was regressed on career stage, competitive style, loss by points or judge's decision, and victory as well as loss by knock-out. The results for this model appear in Table 2. Findings for career stage and competitive style were not reported in this table due to a lack of impact for explaining variance in winning record for this model (Supplementary File C).

The overall model explained a statistically significant and moderate amount of the variance in winning record. The findings from the moderation analyses indicated that the model explained a statistically significant amount of variance in winning record for regional/state (moderate) and amateur (large) athletes only and for following combat sports retained in the model—MMA, MT/KB, and boxing (moderate effects)—and large—for amateurs—amount of variance in winning record for two of the three levels of athletes; and explained a non-significant amount of the variance in winning record for elite athletes. The model explained a large amount of variance in winning record for three of the primary sports—MMA, MT/KB, and boxing—but explained a non-significant portion of the variance in winning record for K & T.

Three broad unique effects were observed in this model. Overall, athletes who had a better record reported fewer losses by points or judge's decision (large effect), with this statistically significant negative effect restricted to regional/state athletes (moderate effect) and amateurs (large effect) athletes as well as the following combat sports—MMA (moderate effect) and MT/KB as well as boxing (large effects). Overall, athletes with a better record reported more victories by knockout, with this effect limited to MMA (moderate effect). Overall, athletes with a better record reported fewer losses by knockout (moderate effect), with moderate effects noted for amateur and regional/state athletes only; and for the following sports—MT/KB (small effect), boxing (moderate effect), and MMA (large effect).

**Model 3 .**

Winning record based on victory and loss by points, disqualification and submission or pin—for MMA, BJJ, Wrestling, and Judo

After the preliminary analysis for this model (Supplementary File D), winning record was regressed on career stage, competitive style, frequency of *non-combat training* sessions each week *without* a competition in the near term, frequency of *non-combat* training sessions each week *with* a competition in the near term, loss by points or judge's decision, and loss by submission or pin (Table 3). Findings for frequency of noncombat training sessions with a competition in the near term were not reported in this table due to a lack of impact for explaining variance in winning record for this model (Supplementary File D).

The overall model explained a statistically significant but small amount of variance in winning record. The moderation analyses indicated that the model explained a statistically significant amount of variance in winning record for amateurs and regional/state athletes (moderate) and for some of the combat sports retained in the model—BJJ (moderate) and wrestling (large).

Three broad unique effects were observed. Athletes who reported a better winning record also reported being at a later stage of their career (small effect). This effect was observed for regional/state (small effect) and amateur (moderate effect) athletes; and for BJJ athletes only (small effect).

Athletes with a better record also reported a more aggressive competitive style (small effect). No moderation effects were noted for this effect across competitive levels or combat sport.

Athletes with a better record also reported fewer losses by submission or pin (moderate effect). This effect was limited to amateurs and regional/state athletes (moderate effects) and to BJJ as well as wrestling athletes.

Two unique findings were noted for this model. Amateurs (moderate effect) and wrestlers (moderate effect) with a better winning record also reported fewer losses by points or judge's decision. BJJ athletes who reported a better winning record also reported a greater frequency of non-combat training sessions each week without a competition in the near term.

**Model 4 .**

Winning record based on victory and loss by points, disqualification and throws and TF—for Wrestling and Judo

After the preliminary analysis for this model (Supplementary File E), winning record was regressed on frequency of *non-combat* training sessions each week *with* a competition in the near-term, victory by throws or TF, loss by throws or TF, loss by points or judge's decision, and loss by submission or pin (Table 4). Note that either (but not both) frequency of *non-combat* training sessions each week *with* or *without* a competition in the near-term could have used as a predictor in this model.

The overall model explained a statistically significant and moderate amount of variance in winning record. The moderation analyses indicated no differences in findings across the different levels of combat sport participation. When examined separately, it was found that the model explained a statistically significant amount of variance in winning record for both wrestling and judo (large).

Four broad unique effects were observed. Athletes with better records reported fewer losses by points or judge's decisions. Athletes with a better record also reported more wins by throws or TF (moderate effect), with the size of the effect (moderate) consistent for both wrestling and judo. Athletes with a better record also reported and fewer losses by throws or TF (moderate effect), with a negative effect of loss by throws or TF reported in judo (large effect). Athletes who reported a better record also reported more losses by points or judge's decision. Athletes with a better winning record also reported fewer losses by submission or pin (small effect), with the size of the effect (small) consistent for wrestling and judo.

One specific finding was observed for this model. While wrestlers who reported a better winning record reported doing less *non-combat* training sessions each week *with* a competition in the near-term, this effect was reversed in judo.

## 4. Discussion

Loss via points or judge's decision generally had a consistent impact on competitive performance (i.e., winning record) across the models. Loss by points or judge's decision had a negative effect on competitive performance overall in model one (small effect), and specifically for amateurs, MMA, MT/KB, and boxing (moderate to large effects); for model two overall (moderate effect), and specifically for amateurs, regional/state, MMA, MT/KB, and boxing (moderate to large effects). In model 3, the negative effect of loss of points or judge's decision on competitive performance was limited to amateurs. While findings from moderation analyses indicate the negative impact of loss by points or judge's decision on overall competitive performance is not universal, taken together, these results reliably show that a greater proportion of losses by points or judge's decision is overall a negative for an athletes' career. Interestingly, perhaps unexpectedly, victories by points were not found to be significantly related to career winningness in any model. Also, surprisingly, a finding inconsistent with our observed trends about losing by points or judge's decision was that for wrestling and judo, a consistent positive association was found in the model between loss by points or judge's decision and competitive performance. Given the questions in this study were framed about proportions of losses, a potential explanation is that grapplers who have more losses by points or judge's decision would have less losses by finishing sequence (such as a pin or throw). Thus, wrestlers and judokas with a greater portion of their losses by decision or points may demonstrate greater defence to bout finishing sequences, or perhaps take less risks that could have their bouts end via loss to finishing sequence. These findings overall may demonstrate for most combat sports aiming to win via points or judge's decision is not an ideal strategy, as it is not as effective a path to career winningness but runs the risk of being detrimental to a career to lose by points or judge's decision. Instead, combat sport athletes should be prioritising and training on paths to victory that avoid judge's decision or points altogether.

Loss via finishing sequence was also a consistent predictor of poorer career winningness. These results were observed in models of loss by knockout (Table 2), loss by submission or pin (Tables 3 and 4), and loss by throws or TF (Table 4). In fact, in models including grappling only sports, losing via finishing sequence was found to have a greater negative relationship to performance than losing via points or judge's decision (Tables 3 and 4). These results combined with the observed positive effect of a greater proportion of losses via points or judge's decision seem to support the proposition that defence to finishing sequences in grappling sports is of paramount importance, and it is preferable to lose via points or judge's decision. Though interestingly in model 2, loss by knock-out, while still significant, did not have as large a negative effect on career winningness than having a greater proportion of losses via points or judge's decision. However, when looking at individual sports, this did not seem to be the case for MMA. The reasoning behind these findings is currently unclear and would be an interesting topic for future research.

Our findings indicate that, offensively, a significant component of combat sports bouts is the potential for finishing sequences such as by submissions or knock-outs. Victory by throws or TF was found to have a positive and moderately-sized association overall and for both wrestling, and judo (Table 4). These results do align with previous research outlining that athletes with greater offensive output finished higher within grappling tournaments [22], and within wrestling, at an emphasis on single leg takedown offence [23]. Victory by knock-out was found to have a positive but small association with career winningness overall, and a more pronounced affect in MMA (Table 2). Such a finding fits within the findings of previous research to show offensive output to be related to increased winningness in striking sports [24, 25]. However, our findings may suggest that beyond simple output alone, it is preferable to use such output to win via a finishing sequence than points or judge's decision. Potentially due to the definitive nature of a finishing sequence, which could even steal a victory despite being significantly down on points. The importance of finishing sequences aligns with previous research outlining the significant strategies for winning in combat sports [8, 26]. However, generally methods of losing showed greater relationships to career winningness compared with methods of winning. Taken together, these results may be used to suggest that sound defence is more important than offence within combat sports. Such results stand to reason as if an athlete demonstrates poor defence their bout can come to an immediate end, whereas solid defence allows them a continuing chance to win the bout via finishing sequence or, points or judge's decision.

Regarding training habits, it is worth noting that frequency of *combat* sport training sessions each week *with or without* a competition in the near-term and frequency of *non-combat* training sessions each week *with or without* a competition in the near term were strongly correlated but not so strongly to be the source of multicollinearity issues in the models. Bearing this in mind, frequency of non-combat and combat training with or without a competition in the near term played a relatively small role in the modelling of competitive winningness as the measures did not make an overall notable contribution to the prediction of competitive winningness, which aligns with previous research in judo finding training volume to not be a predictor of winningness [27], though there may be benefits of increased training outside of winning competitions such as greater bone mineral density which has been observed in wrestlers with higher training loads [28]. Within our study, some specific findings were noted, with frequency of non-combat training sessions each week without a competition in the near term positively and at least moderately associated with competitive winningness for BJJ (Tables 1 and 3) and judo (Table 4). As previous research has reported BJJ to be on the lower end of the spectrum for frequency of non-combat sports training sessions compared to other sports [1], it is possible that our results indicate that better strength and conditioning gives a significant performance edge. Surprisingly, frequency of non-combat sessions was negatively associated (small effect) with competitive winningness for wrestling (Table 4). Considering previous research has reported wrestlers completing more non-combat sport training sessions than other combat sports, these results may indicate that such a high training volume puts them at higher risk of overtraining [1]. This may also be related to a lack of periodisation within training loads, which previous research has observed in MMA [13]. Indeed, future research should be conducted to examine training load planning within wrestling. Training load may also have a significant relationship to injury rate, for example, higher training load has been associated with more frequent injuries in taekwondo [29], though injuries were not assessed within the current study so no conclusion can be drawn in such areas with this data. Outside of these reported differences, frequency of training had no influence on competitive winningness in any combat sport. A potential explanation for this is the general uniformity of training habits across and within combat sports masking any potential benefits of the small percentage that might train differently [1]. Additionally, the nature of our questionnaire did not determine the specifics of the sessions. As such, future research would do well to conduct more in-depth investigations around different approaches to conditioning and their influences on competitive winningness.

Of the demographic measures, personal measures (age, gender, and BMI) were unrelated to competitive performance. However, some sport-specific demographic measures were related to competitive performance. A more aggressive competitive stye had a small positive effect on a winning record where the effect of points and judge's decision was included in the model (Table 1) for amateurs, regional/states, and BJJ, and where submission or pin was possible method of victory or loss (Table 3). These results appear to align with previous research finding a greater volume of strikes landed and takedowns (in MMA) executed were predictors of winningness in MMA and boxing [8, 9]. Possibly athletes who fight more aggressively are more likely to be looked at favourably by judges or put themselves in favourable positions to execute finishing sequences. Being at a later stage of their competitive career had a positive impact on winning record where submission or pin was a possible method of victory or loss (Table 3) for amateurs, regional/state, and BJJ, which likely is an indication that competitive experience is an essential part of competitive winningness. Future research should investigate further into how aggression and competitive experience may influence winningness in combat sports in study designs that analyse the actual results of competitions.

While this study had strengths, such as the large sample size collected and in-depth questionnaire used, there are several limitations that must be considered. There are potential issues with relying on self-reported data, though we did attempt to mitigate this by recruiting a sufficiently large sample. It is important to note that due to small sample sizes within certain groups, some sports needed to be combined for data analysis. Such combinations were done in situations where sports shared a lot of similarities but caution still needs to be used when trying to draw conclusions about specific sports within a grouping. Due to sample size, we have more confidence in the overall findings as well as findings for amateurs and regional/state level, at least for models one to three, with the least confidence in findings related to elite athletes, MMA, Judo and K & T. Also, victory or loss by disqualification was not examined due to insufficient sample size so no conclusions can be drawn in this area. The precision of the competitive records would have varied across combat sports, certain sports like MMA would have found it easier to recall specific records compared to grappling sports like judo where an athlete may have hundreds of bouts. Athletes were asked to give their best guess if they were unsure, but the results to need to be interpreted cautiously due to this. Finally, it is important to consider that simply because correlation was observed, does not mean that there is inherit causation. Combat sports are complicated with many factors that may influence the results that we were unable to control for. The results of this study should be used to make estimates of general trends and be utilised in conjunction with other research and practical experience in the field.

## 5. Conclusion

The purpose of this research was to investigate competitive records across a range of combat sports and try to determine the trends of more winning athletes. The primary findings outlined the importance of combat sport athletes focusing on developing styles that emphasize the ability to win bouts via finishing sequence prior to the full match time concluding. However, it is important to balance the risk of such a style, given the large negative effect that large proportions of losses via finishing sequences can have on a career. Future research would benefit from examining the records of combat sports athletes using collected data as opposed survey data to determine if the trends observed in this study remain important. The results from this study can help inform athletes and coaching staff developing effective strategies for competitions across careers.

## Figures and Tables

**Table 1:** Statistically significant findings for Model 1: winning record based on victory and loss by points as well as disqualification—for all sports.

	Competitive style	Frequency of *noncombat* training sessions each week *without* a competition in the near term	Loss by points or judge's decision	*R* ^2^ (*p*)
	*β*	Β	Β	
*Overall*				
(*n* = 280)	0.18		−0.25	0.11 (0.02)

*Level of competition*				
Amateurs (*n* = 106)	0.20		−0.44	0.25 (<0.01)
Regional/state (*n* = 94)	0.23			0.19 (0.01)
Elite (*n* = 80)^*∗*^				

*Primary sport*				
Mixed martial arts (*n* = 27)^*∗*^				
Muay Thai/Kickboxing (*n* = 43)			−0.64	0.43 (<0.01)
Boxing (*n* = 57)			−0.54	0.29 (<0.01)
Brazilian Jiu Jitsu (*n* = 56)	0.26	0.58		0.27 (<0.01)
Wrestling (*n* = 42)			0.40	0.34 (<0.01)
Judo (*n* = 26)				
Traditional striking sports (*n* = 29)^*∗*^				

*Note*. ^*∗*^While some statistically significant findings for specific predictors for these groups were observed, they were not reported in this table because the variance explained by the model as applied to these groups was not statistically significant.

**Table 2:** Statistically significant findings for Model 2: winning record based on victory and loss by points, disqualification and knock-out—for MMA, MT/KB, boxing, and K and T.

	Loss by points or judge's decision	Victory by knock-out	Loss by knock-out	*R* ^2^ (*p*)
	*β*	*β*	*β*	
*Overall*				
(*n* = 156)	−0.50	0.14	−0.36	0.37 (<0.01)

*Level of competition*				
Amateurs (*n* = 84)	−0.58		−0.44	0.53 (<0.01)
Regional/state (*n* = 29)	−0.48		−0.32	0.29 (<0.01)
Elite (*n* = 43)				

*Primary sport*				
Mixed martial arts (*n* = 27)	−0.43	0.33	−0.50	0.48 (<0.01)
Muay Thai/Kickboxing (*n* = 43)	−0.66		−0.27	0.48 (<0.01)
Boxing (*n* = 57)	−0.56		−0.41	0.44 (<0.01)
Traditional striking sports (*n* = 29)				

**Table 3:** Statistically significant findings for Model 3: winning record based on victory and loss by points, disqualification and submission or pin—for MMA, BJJ, wrestling, and judo.

	Career stage	Competitive style	Frequency of *non-combat* training sessions each week *without* a competition in the near term	Loss by submission or pin	Loss by points or judge's decision	*R* ^2^ (*p*)
	*β*	*β*	Β	*β*	*β*	
*Overall*						
(*n* = 151)	0.20	0.17		−0.31		0.18 (<0.01)

*Current level*						
Amateurs (*n* = 37)	0.43			−0.48	−0.41	0.29 (0.02)
Regional/state (*n* = 69)	0.28			−0.41		0.36 (<0.01)
Elite (*n* = 45)^*∗*^						

*Primary sport*						
Mixed martial arts (*n* = 27)						
Brazilian jiu jitsu (*n* = 56)	0.23		0.46	−0.29		0.39 (<0.01)
Wrestling (*n* = 42)				−0.32	0.30	0.42 (<0.01)
Judo (*n* = 26)						

*Note*. ^*∗*^While some statistically significant findings for specific predictors for this group were observed, they were not reported in this table as the *R*^2^ for the model as applied to this group was not statistically significant.

**Table 4:** Statistically significant findings for Model 4: winning record based on victory and loss by points, disqualification and Ippon, Waza-ari—for wrestling and judo.

	Frequency of *non-combat* training sessions each week *with* a competition in the near-term	Victory by Ippon, Waza-arior technical fall	Loss by Ippon, Waza-ari or technical fall	Loss by points or judge's decision	Loss by submission or pin	*R* ^2^ (*p*)
	*β*	*β*	Β		*β*	
*Overall*						
(*n* = 68)		0.47	−0.36	0.27	−0.26	0.39 (<0.01)

*Primary sport*						
Wrestling (*n* = 42)	−0.23	0.41			−0.26	0.52 (<0.01)
Judo (*n* = 26)	0.33	0.44	−0.63		−0.29	0.59 (<0.01)

## Data Availability

The data are not publicly available but can be provided on reasonable request.

## Supplementary Materials

Supplementary Materials Supplementary File A: This describes the initial screening of the data and provides context on how the models were designed for those who are interested in the statistic methodology. Supplementary Files B, C, D, and E: These appendices provide the full context of the models. In this study, we only present the significant differences, not the nonsignificant findings. All these nonsignificant findings should be available for those who wish to see them in their entirety but there is no realistic way to present these data in the paper itself while keeping the manuscript concise.
