# Supplementary material for: Different Methods of Winning, Losing, and Training in Combat Sports and Their Relationship with Overall Competitive Winningness
Source: Transl Sports Med. 2024 Feb 21;2024:5531981. doi: 10.1155/2024/5531981 (PMC11023722; doi:10.1155/2024/5531981)
Supplement: Supplementary Materials — Supplementary File A: This describes the initial screening of the data and provides context on how the models were designed for those who are interested in the statistic methodology. Supplementary Files B, C, D, and E: These appendices provide the full context of the models. In this study, we only present the significant differences, not the nonsignificant findings. All these nonsignificant findings should be available for those who wish to see them in their entirety but there is no realistic way to present these data in the paper itself while keeping the manuscript concise. [file 5531981.f1.docx]

**Supplementary file A**

**List of Survey Questions**

Q1. What year were you born?

*Dropdown list from 1920-2001*

Q2. What is your sex?

*Male or female*

Q3. How tall are you? (Please specify your unit of measurement such as centimetres or feet)

*Text entry*

Q4. How much do you currently weigh? (Please specify your unit of measurement such as kilograms or pounds)

*Text entry*

***Page break***

Q5. At what age did you begin training combat sports? (Years)

*Dropdown list from 4-70*

Q6. At what age did you begin to compete in combat sports? (Years)

*Dropdown list from 4-70*

Q7. How many times do you train per week without a competition coming up?

Two d*ropdown lists (first is combat sports sessions and second is other forms of training (i.e. strength and conditioning) scale points range from 1-14+*

Q8. How many times do you train per week with a competition coming up?

Two d*ropdown lists (first is combat sports sessions and second is other forms of training (i.e. strength and conditioning) scale points range from 1-14+*

***Page break***

Q9. Which of the previous combat sports have you previously competed in? (Check all that apply)

*Multi-choice matrix with Mixed Martial Arts, Muay Thai/Kickboxing, Boxing, Brazilian Jiu Jitsu, Wrestling, Judo, Karate or Thai Kwon Do.*

Q10. Which one of these combat sports is your current primary combat sport? (i.e. the one most of your training within the last 12 months was in preparation for)

*Multi-choice (only can be checked) matrix with Mixed Martial Arts, Muay Thai/Kickboxing, Boxing, Brazilian Jiu Jitsu, Wrestling, Judo, Karate or Thai Kwon Do.*

***Page break***

S1. Please answer all following questions for your previously indicated primary combat sport only.

**Page break**

Q11. Please describe what levels of competition you have competed at? (Note: some levels may not be applicable to your sport, in such a case, please enter "never participated")

*Matrix table with competitive levels (Regional/state level, National/international level, amateur, semi-professional and professional). Scale points are participated, for a championship and never participated.*

Q12. Which one of these competitive levels is your current primary level of competition? (i.e. the one most of your training within the last 12 months was in preparation for)

*Multi-choice single answer with Regional/state level, National/international level, amateur, semi-professional and professional.*

**Page break**

S2. Please answer all following questions for your primary level of competition only.

**Page break**

Q13. At what stage are you in your competitive career? (please estimate)

*A visual analogue scale ranging from 0-100 with “start of career” at 0, “middle of career” at 50 and “end of career” at 100.*

Q14. Please describe your current competitive record (If unsure , please estimate).

*Dropdown likehert scale with “Wins (total)”, “Losses (total) and “Draws or no contests (total)”. Ranging from 0-300 and 300+*

Q15. How would you describe your competitive style?

*A visual analogue scale ranging from 0-100 with “Defensive” at 0 and “aggressive” at 100.*

Q16. Please describe number of victories you have had by different methods (If the method of victory does not apply to your sport, please select "not applicable")

*Dropdown likert scale with points or judge’s decision, Knock-out, technical knock-out or corners/doctor’s stoppage, submission or pin/technical fall, disqualification, ippon or Waza-ari. Scale includes all of my victories, most of my victories, some of my victories, a few of my victories, none of my victories and not applicable.*

Q17. Please describe number of losses you have had by different methods (If the method of loss does not apply to your sport, please select "not applicable")

*Dropdown likert scale with points or judge’s decision, Knock-out, technical knock-out or corners/doctor’s stoppage, submission or pin/technical fall, disqualification, ippon or Waza-ari. Scale includes all of my losses, most of my losses, some of my losses, a few of my losses, none of my losses and not applicable.*

**Page break**

Q18. Below is a series of questions to measure some of the ways in which you act and think. Read each statement and select the appropriate option. Do not spend too much time on any statement. Answer quickly and honestly.

*A multiple-choice matrix with the scale points “completely agree”, “disagree”, “neutral”, “agree” and “completely agree”. The questions included are:*

- *I am often confused about what emotion I am feeling*
- *It is difficult for me to find the right words for my feelings.*
- *I have physical sensations that even doctors don't understand.*
- *I am able to describe my feelings easily.*
- *I prefer to analyze problems rather than just describe them.*
- *When I am upset, I don't know if I am sad, frightened, or angry.*
- *I am often puzzled by sensations in my body.*
- *I prefer to just let things happen rather than to understand why they turned out that way.*
- *I have feelings that I can't quite identify.*
- *Being in touch with emotions is essential.*
- *I find it hard to describe how I feel about people.*
- *People tell me to describe my feelings more.*
- *I don't know what's going on inside me.*
- *I often don't know why I am angry.*
- *I prefer talking to people about their daily activities rather than their feelings.*
- *I prefer to watch "light" entertainment shows rather than psychological dramas.*
- *It is difficult for me to reveal my innermost feelings, even to close friends.*
- *I can feel close to someone, even in moments of silence.*
- *I find examination of my feelings useful in solving personal problems.*
- *Looking for hidden meanings in movies or plays distracts from their enjoyment.*

**Page break**

Q19. Below are some questions to measure some of the ways in which you act and think. Read each statement and select the appropriate option. Do not spend too much time on any statement. Answer quickly and honestly.

*A multiple-choice matrix with the scale points “rarely/never”, “occasionally”, “often” and “almost always/always”. The questions included are:*

- *I plan tasks carefully.*
- *I do things without thinking.*
- *I make-up my mind quickly.*
- *I am happy-go-lucky.*
- *I don't "pay attention."*
- *I have "racing" thoughts.*
- *I plan trips well ahead of time.*
- *I am self-controlled.*
- *I concentrate easily.*
- *I save regularly.*
- *I "squirm" at plays or lectures.*
- *I am a careful thinker.*
- *I plan for job security.*
- *I say things without thinking.*
- *I like to think about complex problems.*
- *I change jobs.*
- *I act on impulse.*
- *I get easily bored when solving thought problems.*
- *I act on the spur of the moment.*
- *I am a steady thinker.*
- *I change residences.*
- *I buy things on impulse.*
- *I can only think about one thing at a time.*
- *I change hobbies.*
- *I spend or charge more than I earn.*
- *I often have extraneous thoughts when thinking.*
- *I am more interested in the present than the future.*
- *I am restless at the theater or lectures.*
- *I like puzzles.*
- *I am future oriented.*

Q20. Below is another series of questions to measure some of the ways in which you act and think. Read each statement and select the appropriate option. Do not spend too much time on any statement. Answer quickly and honestly.

Some questions mention bad or unpleasant emotions, this means emotions like sadness, anger, or fear. Some questions mention good or pleasant emotions, this means emotions like happiness, amusement, or excitement.

*A multiple-choice matrix with the scale points “strongly disagree”, “disagree”, “somewhat disagree”, “nether agree not disagree”, “somewhat agree”, ‘agree” and “strongly agree”. The questions included are:*

- *When I’m feeling bad (feeling an unpleasant emotion), I can’t find the right words to describe those feelings.*
- *When I’m feeling bad, I can’t tell whether I’m sad, angry, or scared.*
- *I tend to ignore how I feel.*
- *When I’m feeling good (feeling a pleasant emotion), I can’t find the right words to describe those feelings.*
- *When I’m feeling good, I can’t tell whether I’m happy, excited, or amused.*
- *I prefer to just let my feelings happen in the background, rather than focus on them.*
- *When I’m feeling bad, I can’t talk about those feelings in much depth or detail.*
- *When I’m feeling bad, I can’t make sense of those feelings.*
- *I don’t pay attention to my emotions.*
- *When I’m feeling good, I can’t talk about those feelings in much depth or detail.*
- *When I’m feeling good, I can’t make sense of those feelings.*
- *Usually, I try to avoid thinking about what I’m feeling.*
- *When something bad happens, it’s hard for me to put into words how I’m feeling.*
- *When I’m feeling bad, I get confused about what emotion it is.*
- *I prefer to focus on things I can actually see or touch, rather than my emotions.*
- *When something good happens, it’s hard for me to put into words how I’m feeling.*
- *When I’m feeling good, I get confused about what emotion it is.*
- *I don’t try to be ‘in touch’ with my emotions.*
- *When I’m feeling bad, if I try to describe how I’m feeling I don’t know what to say.*
- *When I’m feeling bad, I’m puzzled by those feelings.*
- *It’s not important for me to know what I’m feeling.*
- *When I’m feeling good, if I try to describe how I’m feeling I don’t know what to say.*
- *When I’m feeling good, I’m puzzled by those feelings.*
- *It’s strange for me to think about my emotions.*

**Page break**

Q21. Have you ever lost weight to compete before?

*Multiple-choice with “yes” or “no”.* ***If “no” is selected, then the survey will end.***

Q22. Would you be willing to take part in our brief (5 minutes) survey on weight-loss practices?

*Multiple-choice with “yes” or “no”.* ***If “no” is selected, then the survey will end.***

Q23. How much do you weigh without a competition coming up? (Please specify your unit of measurement such as kilograms or pounds)

*Text entry box.*

Q24. In the last 2 years, how often do you lose weight for a competition?

*A dropdown box with “every competition”, “most competitions”, “some competitions”, “a few competitions” and “no competitions”*

Q25. At what age did you begin losing weight for competitions? (years old)

*A dropdown box including “under 10” and then numbers ranging from 10-80*

Q26. How much weight would you typically lose for a competition? (Please specify your unit of measurement such as kilograms or pounds)

*Text entry box.*

Q27. What is the most amount of weight you have lost for a competition? (Please specify your unit of measurement such as kilograms or pounds)

*Text entry box.*

Q28. How much weight do you typically lose in the two weeks before the weigh-in? (Please specify your unit of measurement such as kilograms or pounds)

*Text entry box.*

Q29. Of that weight, how much is lost within 24 hours of the weigh-in? (Please specify your unit of measurement such as kilograms or pounds)

*Text entry box.*

Q30. How much weight do you typically regain by the competition? (Please specify your unit of measurement such as kilograms or pounds)

*Text entry box.*

Q31. How many hours were between the weigh-in and the competition at your last competition?

*Text entry box.*

**Page break**

Q32. The table below presents several methods of rapid weight loss. Using the table below, how often do you use each of the following methods before a competition? (check all items)

*A multiple-choice matrix with the scale points “always”, “sometimes”, “almost never”, “never used” and “I don’t use anymore”. The questions included are:*

- *Gradual dieting (using more than 2 weeks)*
- *Skipping 1 or 2 meals*
- *Fasting (not eating all day)*
- *Restricting fluid ingestion*
- *Increased exercise (more than usual)*
- *Training in heated rooms*
- *Sauna*
- *Plastic/rubber suits or towel wrapping*
- *Use winter or plastic suits for whole day (without exercising)*
- *Spitting*
- *Laxatives*
- *Diuretics*
- *Diet Pills*
- *Vomiting*
- *Excessive fluid ingestion (water loading)*

**Supplementary file B**

**Details of initial data screening**

As a result of initial data screening for normality (skewness > 2 and kurtosis > 7 – see West, Finch, & Curran, 1995) – the two highest categories (i.e., ‘most’ and ‘all’) were recoded as a single category (i.e., ‘most or all’) for four methods of victory or loss – loss according to points or judge’s decisions, victory by knockout, technical knockout, or corner / doctors’ stoppage (referred hereafter as ‘knockout’), and victory or loss by submission or pin.

Most responses for the other five methods of victory or loss were found for the lowest category (‘i.e., none): loss by knockout (*n*= 152, or 75%); victory by disqualification (*n*= 166, or 83%); loss by disqualification (*n*= 177, or 89%); victory by Ippon, Waza-ari or technical fall (*n*= 43, or 39%); and loss by Ippon, Waza-ari or technical fall (*n*= 58, or 53%).

.

**Supplementary file C: Preliminary Analysis for Model 1**

Step 1: Correlation matrix for the initial standard multiple regression analysis (*n*=280).

|  | 1 | 2 | 3 | 4 | 5 | 6 | 7 | 8 | 9 | 10 | 11 | 12 | 13 | 14 |
| --- | --- | --- | --- | --- | --- | --- | --- | --- | --- | --- | --- | --- | --- | --- |
|  |  |  |  |  |  |  |  |  |  |  |  |  |  |  |
| 1. Winning record | 1.00 |  |  |  |  |  |  |  |  |  |  |  |  |  |
| 2. Age | -.02 | 1.00 |  |  |  |  |  |  |  |  |  |  |  |  |
| 3. Gender | .00 | .02 | 1.00 |  |  |  |  |  |  |  |  |  |  |  |
| 4. BMI | -.05 | .36* | .19* | 1.00 |  |  |  |  |  |  |  |  |  |  |
| 5. Career stage | .03 | .51* | .11 | .35* | 1.00 |  |  |  |  |  |  |  |  |  |
| 6. Competitive style | .19* | .12* | .00 | -.01 | .04 | 1.00 |  |  |  |  |  |  |  |  |
| 7. Frequency of *combat* sport training sessions each week *without* a competition in the near-term | .01 | -.08 | -.12 | -.04 | -.06 | .18 | 1.00 |  |  |  |  |  |  |  |
| 8. Frequency of *non-combat* training sessions each week *without* a competition in the near term | .09 | .05 | .07 | .08 | .06 | .07 | .06 | 1.00 |  |  |  |  |  |  |
| 9. Frequency of *combat* sport training sessions each week *with* a competition in the near-term | .06 | -.06 | -.06 | -.08 | -.07 | .22* | .72 | .06 | 1.00 |  |  |  |  |  |
| 10. Frequency of *non-combat* training sessions each week *with* a competition in the near-term | .04 | .03 | -.02 | .01 | .04 | .09 | .08 | .68 | .14* | 1.00 |  |  |  |  |
| 11. Victory by points or judge's decision | .02 | -.01 | -.17* | -.10 | .13* | -.08 | -.09 | -.01 | -.06 | .03 | 1.00 |  |  |  |
| 12. Loss by points or judge's decision | -.26* | .04 | -.11 | .00 | .14* | -.04 | .08 | .01 | .04 | -.01 | .32* | 1.00 |  |  |
| 13. Victory by disqualification | .00 | .00 | .02 | .05 | .19* | .04 | -.01 | .04 | -.02 | -.01 | -.11* | -.06 | 1.00 |  |
| 14. Loss by disqualification | .06 | .14* | .01 | .14* | .14* | .14* | .00 | .11* | -.01 | .02 | -.09 | .02 | .43* | 1.00 |

Note: * *p*< .05.

Step 2: Results for the initial standard multiple regression analysis (*n*=280).

| *R*^2^= .14, *p*< .01 | B | SE | 95% CI | *p* | β | *r* with winning record | *p* | Tolerance | VIF | *M* | *SD* |
| --- | --- | --- | --- | --- | --- | --- | --- | --- | --- | --- | --- |
| Winning Record |  |  |  |  |  |  |  |  |  | 67.25 | 21.77 |
| (Constant) | 71.19 | 11.01 | 49.51; 92.87 | <.01 |  |  |  |  |  |  |  |
| Age | -.19 | .16 | -.51; .13 | .24 | -.08 | -.02 | .37 | .66 | 1.51 | 28.36 | 9.39 |
| Gender | -1.81 | 3.92 | -9.53; 5.90 | .64 | -.03 | .00 | .49 | .89 | 1.13 | .87 | .34 |
| BMI | -.27 | .34 | -0.95; 0.40 | .43 | -.05 | -.05 | .22 | .78 | 1.28 | 25.11 | 4.11 |
| Career stage | .08 | .05 | -.013; .17 | .09 | .12 | .03 | .29 | .63 | 1.59 | 46.56 | 32.72 |
| **Competitive style** | **.18** | **.06** | **.06; .30** | **<.01** | **.18** | **.19** | **<.01** | **.91** | **1.11** | **62.58** | **21.36** |
| Frequency of *combat* sport training sessions each week *without* a competition in the near-term | -.54 | .98 | -2.46; 1.39 | .59 | -.05 | .01 | .41 | .46 | 2.16 | 4.14 | 1.86 |
| Frequency of *non-combat* training sessions each week *without* a competition in the near term | 1.41 | .86 | -0.28; 3.10 | .10 | .13 | .09 | .07 | .51 | 1.95 | 2.99 | 2.01 |
| Frequency of *combat* sport training sessions each week *with* a competition in the near-term | .70 | .77 | -0.80; 2.21 | .36 | .08 | .06 | .15 | .47 | 2.15 | 5.44 | 2.38 |
| Frequency of *non-combat* training sessions each week *with* a competition in the near-term | -.82 | .80 | -2.39; 0.75 | .30 | -.08 | .04 | .26 | .51 | 1.95 | 3.29 | 2.17 |
| Victory by points or judge's decision | 1.70 | 1.04 | -0.34; 3.74 | .10 | .10 | .02 | .40 | .81 | 1.23 | 3.30 | 1.33 |
| **Loss by points or judge's decision** | **-4.30** | **.85** | **-5.98; -2.61** | **<.01** | **-.31** | **-.26** | **<.01** | **.87** | **1.16** | **3.19** | **1.56** |
| Victory by disqualification | -4.18 | 4.19 | -12.44; 4.08 | .32 | -.07 | .00 | .48 | .76 | 1.32 | .13 | .34 |
| Loss by disqualification | 4.98 | 4.96 | -4.789; 14.75 | .32 | .07 | .06 | .17 | .76 | 1.31 | .09 | .29 |

Note: Values in bold, *p*< .05. VIF= Variance Inflation Factor.

**Observations and decisions**

The following variables were retained as they were statistically significant contributors to explaining variance in winning record: competitive style and loss by points or judge’s decision.

Step 3: Moderation analyses.

|  | Competitive Level (*df*= 2) | Primary Sport (*df*= 6) |
| --- | --- | --- |
| Age | χ^2^= 0.97, *p*= .61 | χ^2^= 5.74, *p*= .45 |
| Gender | χ^2^= 1.57, *p*= . 46 | χ^2^= 12.83, *p*= .05 |
| BMI | χ^2^= 1.57, *p*= . 46 | χ^2^= 3.94, *p*= .69 |
| Career stage | χ^2^= 3.31, *p*= .19 | χ^2^= 9.58, *p*= .14 |
| Competitive style | χ^2^= 2.70, *p*= .26 | χ^2^= 0.65, *p*= .99 |
| Frequency of *combat* sport training sessions each week *without* a competition in the near-term | χ^2^= 0.74, *p*= .69 | χ^2^= 6.31, *p*= .39 |
| **Frequency of *non-combat* training sessions each week *without* a competition in the near term** | **χ^2^= 7.68, *p*= .02** | **χ^2^= 16.51, *p*= .01** |
| Frequency of *combat* sport training sessions each week *with* a competition in the near-term | χ^2^= 1.18, *p*= .55 | χ^2^= 1.74, *p*= .94 |
| **Frequency of *non-combat* training sessions each week *with* a competition in the near-term** | **χ^2^= 11.89, *p*= .03** | **χ^2^= 24.52, *p*< .01** |
| Victory by points or judge's decision | χ^2^= 1.60, *p*= .45 | χ^2^= 2.26, *p*= .89 |
| **Loss by points or judge's decision** | **χ^2^= 12.60, *p*< .01** | **χ^2^= 36.08, *p*< .01** |
| Victory by disqualification | χ^2^= 0.39, *p*= .82 | χ^2^= 11.47, *p*= .07 |
| Loss by disqualification | χ^2^= 0.17, *p*= .92 | χ^2^= 4.58, *p*= .60 |

Note: Values in bold, *p*< .05.

**Observations and decisions**

Moderation effects for associations with winning record were observed for: frequency of *non-combat* training sessions each week *without* or *with* a competition in the near term (for competitive level and primary sport); and loss by points or judge's decision. Because loss points or judge’s decision had already been retained for the final model, only frequency of *non-combat* training sessions each week *without* or *with* a competition in the near term (for competitive level and primary sport) were added to the model after the moderation analyses.

Step 4: Final model – overall as well as by competitive level and primary sport.

|  | B | SE | 95% CI | *p* | β | *r* with winning record | *p* | Tolerance | VIF | *M* | *SD* |
| --- | --- | --- | --- | --- | --- | --- | --- | --- | --- | --- | --- |
|  |  |  | Overall |  |  |  |  |  |  |  |  |
| Winning Record |  |  |  |  |  |  |  |  |  | 67.25 | 21.77 |
| *n*=280*; R*^2^= .11, *p*= .02 |  |  |  |  |  |  |  |  |  |  |  |
| (Constant) | 65.35 | 4.98 | 55.54; 75.16 |  |  |  |  |  |  |  |  |
| Competitive style | .18 | .06 | .07; .30 | <.01 | .18 | .19 | <.01 | .99 | 1.01 | 62.58 | 21.36 |
| Frequency of *non-combat* training sessions each week *without* a competition in the near term | 1.25 | .84 | -0.41; 2.91 | .14 | .12 | .09 | .12 | .54 | 1.87 | 2.99 | 2.01 |
| Frequency of *non-combat* training sessions each week *with* a competition in the near-term | -.56 | .78 | -2.10; 0.98 | .47 | -.06 | .04 | .07 | .54 | 1.87 | 3.29 | 2.17 |
| Loss by points or judge's decision | -3.55 | .80 | -5.11 -1.98 | <.01 | -.25 | -.26 | <.01 | 1.00 | 1.00 | 3.19 | 1.56 |
| *n*= 27; *R*^2^ = .21, *p=* .13 |  |  | MMA |  |  |  |  |  |  |  |  |
| Competitive style | -0.07 | 0.21 | -0.41; 0.28 | .76 | -.07 |  |  |  |  |  |  |
| Frequency of *non-combat* training sessions each week *without* a competition in the near term | -3.96 | 3.41 | -9.56; 1.65 | .25 | -.44 |  |  |  |  |  |  |
| Frequency of *non-combat* training sessions each week *with* a competition in the near-term | 4.37 | 3.59 | -1.54; 10.27 | .22 | .49 |  |  |  |  |  |  |
| Loss by points or judge's decision | -5.23 | 2.60 | -9.51; -0.94 | **.05*** | **-.40** |  |  |  |  |  |  |
| *n*= 43; *R*^2^ = .43, *p* <.01 |  |  | MT / KB |  |  |  |  |  |  |  |  |
| Competitive style | 0.27 | 0.15 | 0.03; 0.51 | .06 | .22 |  |  |  |  |  |  |
| Frequency of *non-combat* training sessions each week *without* a competition in the near term | 1.23 | 1.74 | -1.64; 4.10 | .48 | .09 |  |  |  |  |  |  |
| Frequency of *non-combat* training sessions each week *with* a competition in the near-term | -1.87 | 1.47 | -4.30; 0.55 | .20 | -.16 |  |  |  |  |  |  |
| Loss by points or judge's decision | -8.01 | 1.49 | -10.52; -5.63 | **<.01** | **-.64** |  |  |  |  |  |  |
|  |  |  |  |  |  |  |  |  |  |  |  |
|  |  |  |  |  |  |  |  |  |  |  |  |
|  |  |  |  |  |  |  |  |  |  |  |  |
| Table (continued) |  |  |  |  |  |  |  |  |  |  |  |
| *n*= 57; *R*^2^ = .29, *p*< .01 |  |  | Boxing |  |  |  |  |  |  |  |  |
| Competitive style | 0.13 | 0.15 | -0.12; 0.38 | .40 | .01 |  |  |  |  |  |  |
| Frequency of *non-combat* training sessions each week *without* a competition in the near term | -0.03 | 1.44 | -2.34; 2.34 | .98 | -.02 |  |  |  |  |  |  |
| Frequency of *non-combat* training sessions each week *with* a competition in the near-term | 1.75 | 1.74 | -1.11; 4.60 | .32 | .13 |  |  |  |  |  |  |
| Loss by points or judge's decision | -8.11 | 1.77 | -11.03; -5.19 | **<.01** | **-.54** |  |  |  |  |  |  |
| *n*= 56; *R*^2^ = .27, *p*< .01 |  |  | BJJ |  |  |  |  |  |  |  |  |
| Competitive style | 0.25 | 0.12 | 0.06; 0.44 | **.03** | **.26** |  |  |  |  |  |  |
| Frequency of *non-combat* training sessions each week *without* a competition in the near term | 8.56 | 2.46 | 4.51; 12.61 | **<.01** | **.58** |  |  |  |  |  |  |
| Frequency of *non-combat* training sessions each week *with* a competition in the near-term | -1.56 | 2.13 | -5.06; 1.94 | .46 | -.12 |  |  |  |  |  |  |
| Loss by points or judge's decision | 1.19 | 1.90 | -1.93; 4.32 | .53 | .08 |  |  |  |  |  |  |
| *n*= 42; *R*^2^ = .34, *p*< .01 |  |  | Wrestling |  |  |  |  |  |  |  |  |
| Competitive style | 0.06 | 0.09 | -0.09; 0.21 | .50 | .01 |  |  |  |  |  |  |
| Frequency of *non-combat* training sessions each week *without* a competition in the near term | -0.13 | 1.45 | -2.58; 2.32 | .93 | -.02 |  |  |  |  |  |  |
| Frequency of *non-combat* training sessions each week *with* a competition in the near-term | -1.75 | 1.34 | -3.40; 0.50 | .20 | -.28 |  |  |  |  |  |  |
| Loss by points or judge's decision | 6.57 | 2.16 | 3.01; 10.12 | **<.01** | **.40** |  |  |  |  |  |  |
| *n*= 26; *R*^2^ = .20 (.16) |  |  | Judo |  |  |  |  |  |  |  |  |
| Competitive style | 0.18 | 0.21 | -0.16; 0.53 | .37 | .19 |  |  |  |  |  |  |
| Frequency of *non-combat* training sessions each week *without* a competition in the near term | 3.47 | 4.11 | -3.28; 10.22 | .40 | .28 |  |  |  |  |  |  |
| Frequency of *non-combat* training sessions each week *with* a competition in the near-term | 1.22 | 3.99 | -5.34; 7.78 | .76 | .10 |  |  |  |  |  |  |
| Loss by points or judge's decision | -0.56 | 3.46 | -6.25; 5.13 | .87 | -.03 |  |  |  |  |  |  |
|  |  |  |  |  |  |  |  |  |  |  |  |
|  |  |  |  |  |  |  |  |  |  |  |  |
| Table (continued) |  |  |  |  |  |  |  |  |  |  |  |
| *n*= 29; *R*^2^ = .27, *p=* .06 |  |  | T&K |  |  |  |  |  |  |  |  |
| Competitive style | 0.29 | 0.16 | 0.03; 0.55 | **.07** | **.30** |  |  |  |  |  |  |
| Frequency of *non-combat* training sessions each week *without* a competition in the near term | 9.50 | 4.18 | 2.61; 16.38 | **.02** | **.67** |  |  |  |  |  |  |
| Frequency of *non-combat* training sessions each week *with* a competition in the near-term | -5.57 | 2.48 | -9.64; -1.49 | **.03** | **-.70** |  |  |  |  |  |  |
| Loss by points or judge's decision | -6.40 | 2.62 | -10.71; -2.08 | **.02** | **-.44** |  |  |  |  |  |  |
| *n*= 106; *R*^2^ = .25, p <.01 |  |  | Amateurs |  |  |  |  |  |  |  |  |
| Competitive style | 0.23 | 0.10 | 0.07; 0.39 | **.02** | **.20** |  |  |  |  |  |  |
| Frequency of *non-combat* training sessions each week *without* a competition in the near term | 0.47 | 1.30 | -1.68; 2.61 | .72 | .04 |  |  |  |  |  |  |
| Frequency of *non-combat* training sessions each week *with* a competition in the near-term | -1.21 | 1.32 | -3.37; 0.96 | .36 | -.10 |  |  |  |  |  |  |
| Loss by points or judge's decision | -6.60 | 1.25 | -8.67; -4.54 | **<.01** | **-.44** |  |  |  |  |  |  |
| *n*= 94; *R*^2^= .19, *p=* .01 |  |  | Regional / State |  |  |  |  |  |  |  |  |
| Competitive style | 0.22 | 0.09 | 0.07; 0.37 | **.02** | **.23** |  |  |  |  |  |  |
| Frequency of *non-combat* training sessions each week *without* a competition in the near term | 2.18 | 1.57 | -0.41; 4.77 | .17 | .18 |  |  |  |  |  |  |
| Frequency of *non-combat* training sessions each week *with* a competition in the near-term | 2.39 | 1.46 | -0.02; 4.80 | .10 | .21 |  |  |  |  |  |  |
| Loss by points or judge's decision | -1.78 | 1.37 | -4.03; 0.47 | .19 | -.12 |  |  |  |  |  |  |
| *n*= 80; *R*^2^= .06, *p=* .26 |  |  | Elite |  |  |  |  |  |  |  |  |
| Competitive style | 0.04 | 0.09 | -0.12; 0.19 | .71 | .04 |  |  |  |  |  |  |
| Frequency of *non-combat* training sessions each week *without* a competition in the near term | 1.67 | 1.21 | -0.33; 3.67 | .17 | .22 |  |  |  |  |  |  |
| Frequency of *non-combat* training sessions each week *with* a competition in the near-term | -2.04 | 1.04 | -3.75; -0.33 | **.05*** | **-.31** |  |  |  |  |  |  |
| Loss by points or judge's decision | -1.19 | 1.24 | -3.22; 0.84 | .34 | -.11 |  |  |  |  |  |  |

Note: Values in bold, *p*< .05. VIF= Variance Inflation Factor.

**Supplementary file D: Preliminary Analysis for Model 2**

Step 1: Correlation matrix for the initial standard multiple regression analysis (*n*=156).

|  | 1 | 2 | 3 | 4 | 5 | 6 | 7 | 8 | 9 | 10 | 11 | 12 | 13 | 14 | 15 | 16 |
| --- | --- | --- | --- | --- | --- | --- | --- | --- | --- | --- | --- | --- | --- | --- | --- | --- |
| 1. Winning Record | 1.00 |  |  |  |  |  |  |  |  |  |  |  |  |  |  |  |
| 2. Age | -.09 | 1.00 |  |  |  |  |  |  |  |  |  |  |  |  |  |  |
| 3. Gender | .07 | -.08 | 1.00 |  |  |  |  |  |  |  |  |  |  |  |  |  |
| 4. BMI | -.12 | .14* | .20* | 1.00 |  |  |  |  |  |  |  |  |  |  |  |  |
| 5. Career Stage | -.12 | .48* | .10 | .18* | 1.00 |  |  |  |  |  |  |  |  |  |  |  |
| 6. Competitive Style | .18* | .07 | -.02 | .01 | -.02 | 1.00 |  |  |  |  |  |  |  |  |  |  |
| 7. Frequency of *combat* sport training sessions each week *without* a competition in the near-term | .02 | -.13 | -.14 | -.07 | -.23* | .19* | 1.00 |  |  |  |  |  |  |  |  |  |
| 8. Frequency of *non-combat* training sessions each week *without* a competition in the near term | .02 | .00 | .02 | .04 | -.11 | .13 | -.05 | 1.00 |  |  |  |  |  |  |  |  |
| 9. Frequency of *combat* sport training sessions each week *with* a competition in the near-term | .05 | -.01 | -.04 | -.10 | -.13* | .24* | .78* | -.03 | 1.00 |  |  |  |  |  |  |  |
| 10. Frequency of *non-combat* training sessions each week *with* a competition in the near-term | .00 | -.01 | -.06 | -.02 | -.06 | .18* | .08 | .59* | .14* | 1.00 |  |  |  |  |  |  |
| 11. Victory by points or judge's decision | -.01 | .09 | -.18* | -.08 | .21* | -.16* | -.21* | -.01 | -.18* | .01 | 1.00 |  |  |  |  |  |
| 12. Loss by points or judge's decision | -.48* | .11 | -.10 | .04 | .17* | -.10 | .06 | .07 | .01 | .01 | .24* | 1.00 |  |  |  |  |
| 13. Victory by knock-out | .16 | .02 | .21* | .07 | -.10 | .18* | .07 | .04 | .13 | .06 | -.76* | -.10 | 1.00 |  |  |  |
| 14. Loss by knock-out | -.28* | .09 | .14* | .03 | .11 | -.05 | -.08 | -.11 | -.05 | -.01 | -.18* | -.13* | .11 | 1.00 |  |  |
| 15. Victory by disqualification | -.11 | .05 | -.09 | .07 | .10 | -.033 | -.09 | -.00 | -.13* | -.09 | -.13 | -.03 | -.05 | .13 | 1.00 |  |
| 16. Loss by disqualification | .03 | .08 | -.06 | .10 | .05 | .07 | -.13* | .13 | -.07 | .04 | .00 | -.02 | .03 | -.045 | .35* | 1.00 |

Note: * *p*< .05.

Step 2: Results for the initial standard multiple regression analysis (*n*=156).

| *R*^2^= .46 (*p*< .01) | B | SE | 95% CI | *p* | β | *r* with winning record | *p* | Tolerance | VIF | *M* | *SD* |
| --- | --- | --- | --- | --- | --- | --- | --- | --- | --- | --- | --- |
| Winning Record |  |  |  |  |  |  |  |  |  | 68.26 | 23.32 |
| (Constant) | 53.43 | 17.10 | 19.63; 87.24 | .01 |  |  | . |  |  |  |  |
| Age | -.08 | .22 | -.51; .36 | .72 | -.03 | -.09 | .14 | .71 | 1.41 | 26.81 | 7.82 |
| Gender | 4.30 | 4.14 | -3.88; 12.49 | .30 | .07 | .07 | .20 | .80 | 1.25 | .81 | .39 |
| BMI | -0.60 | 0.40 | -1.49; .29 | .19 | -.10 | -.12 | .08 | .89 | 1.12 | 24.30 | 3.40 |
| Career Stage | .00 | .06 | -.11; .11 | .99 | .00 | -.12 | .06 | .65 | 1.54 | 41.12 | 31.48 |
| **Competitive Style** | **.10** | **.07** | **-.04; .25** | **.16** | **.10** | **.18** | **.01** | **.87** | **1.15** | **62.73** | **21.294** |
| Frequency of *combat* sport training sessions each week *without* a competition in the near-term | 1.53 | 1.45 | -1.33; 4.39 | .29 | .12 | .02 | .42 | .33 | 3.08 | 4.06 | 1.76 |
| Frequency of *non-combat* training sessions each week *without* a competition in the near term | 0.51 | 0.95 | -1.37; 2.39 | .59 | .04 | .02 | .41 | .59 | 1.69 | 2.79 | 1.97 |
| Frequency of *combat* sport training sessions each week *with* a competition in the near-term | -0.45 | 1.07 | -2.56; 1.67 | .68 | -.04 | .05 | .27 | .35 | 2.87 | 5.45 | 2.29 |
| Frequency of *non-combat* training sessions each week *with* a competition in the near-term | -0.73 | 0.84 | -2.39; 0.94 | .39 | -.07 | .00 | .50 | .60 | 1.67 | 3.28 | 2.21 |
| **Victory by points or judge's decision** | **7.99** | **1.90** | **4.24; 11.73** | **<.01** | **.47** | **-.01** | **.44** | **.31** | **3.25** | **3.59** | **1.38** |
| **Loss by points or judge's decision** | **-7.75** | **0.92** | **-9.57; -5.94** | **<.01** | **-.57** | **-.48** | **<.01** | **.84** | **1.19** | **3.42** | **1.72** |
| Victory by disqualification | 0.47 | 7.15 | -13.67; 14.61 | .95 | .01 | -.11 | .08 | .75 | 1.34 | .06 | .23 |
| Loss by disqualification | 1.02 | 7.71 | -14.22; 16.25 | .90 | .10 | .03 | .36 | .82 | 1.23 | .04 | .21 |
| **Victory by knock-out** | **9.50** | **2.11** | **5.34; 13.67** | **<.01** | **.47** | **.16** | **.02** | **.35** | **2.84** | **2.13** | **1.16** |
| **Loss by knock-out** | **-16.96** | **3.66** | **-24.19; -9.73** | **<.01** | **-.31** | **-.28** | **<.01** | **.88** | **1.14** | **.23** | **.42** |

Note: Values in bold, *p*< .05. VIF= Variance Inflation Factor.

**Observations and decisions**

The statistically significant contributors to explaining variance in winning record were competitive style, victory and loss by points or judge’s decision, and victory and loss by knock-out.

It was decided to retain by victory and loss by knock-out because the correlation (.11) between these variables and winning record was statistically significant. Suppression was observed in the model as the correlation between winning record and victory by points or judge’s decision was not statistically significant but the unique effect of victory by points or judge’s decision on winning record was statistically significant. A likely source of this suppression effect was that, for this subset of combat sport athletes – MMA, BJJ, Wrestling, and Judo – the correlation between victory and victory by knockout was statistically significant and very large (-.76). As the Pearson *r* between winning record and victory by knockout was statistically significant whereas Pearson *r* between winning record and victory by points or judge’s decision was not statistically significant and small, it was decided to retain loss by points or judge’s decision.

Confounding was observed was the correlation between competitive style on winning record was statistically significant but was this variable was not a statistically significant predictor in the regression model for step 1. Hence, competitive style was retained in the model.

Overall, the following variables were initially retained: loss by points or judge's decision, victory as well as loss by knock-out, and competitive style.

Step 3: Moderation analyses.

|  | Competitive Level (*df*= 2) | Primary Sport (*df*= 3) |
| --- | --- | --- |
| Age | χ^2^= 1.60, *p*= .45 | χ^2^= 3.31, *p*= .35 |
| Gender | χ^2^= 0.77, *p*= .68 | **χ^2^= 9.23, *p*= .03** |
| BMI | χ^2^= 0.48, *p*= .78 | χ^2^= 1.21, *p*= .75 |
| Career stage | **χ^2^= 6.44, *p*= .04** | χ^2^= 1.62, *p*= .66 |
| Competitive style | χ^2^= 3.31, *p*= .19 | χ^2^= 0.12, *p*= .99 |
| Frequency of *combat* sport training sessions each week *without* a competition in the near-term | χ^2^= 0.29, *p*= .87 | χ^2^= 3.31, *p*= .35 |
| Frequency of *non-combat* training sessions each week *without* a competition in the near term | χ^2^= 1.82, *p*= .41 | χ^2^= 1.01, *p*= .80 |
| Frequency of *combat* sport training sessions each week *with* a competition in the near-term | χ^2^= 0.67, *p*= .72 | χ^2^= 0.46, *p*= .93 |
| Frequency of *non-combat* training sessions each week *with* a competition in the near-term | χ^2^= 1.22, *p*= .54 | χ^2^= 0.43, *p*= .94 |
| Victory by points or judge's decision | χ^2^= 3.46, *p*= .18 | χ^2^= 1.11, *p*= .78 |
| Loss by points or judge's decision | **χ^2^= 9.24, *p*= <.01** | χ^2^= 1.86, *p*= .60 |
| Victory by disqualification | χ^2^= 4.23, *p*= .12 | χ^2^= 5.05, *p*= .17 |
| Loss by disqualification | Not conducted ^a^ | χ^2^= 2.05, *p*= .56 |
| Victory by knock-out, technical knock-out or corner/doctor's stoppage | χ^2^= 1.23, *p*= .54 | χ^2^= 0.33, *p*= .95 |
| Loss by knock-out, technical knock-out or corner/doctor's stoppage | χ^2^= 5.34, *p*= .07 | χ^2^= 2.15, *p*= .55 |

Note: Values in bold, *p*< .05. ^a^ This analysis was not conducted because one of the three groups had no participants who had lost by disqualification.

**Observations and decisions:**

As moderation effects were observed for career stage, it was retained for the final analysis (bearing in mind that loss by points or judge's decision was retained after step 2).

While a moderation effect for gender was observed, separate analysis by gender for competitive style was not conducted because of the small numbers of female participants for each primary – MMM had three females (of *n*= 27, or 11%); MT / KB had nine females (of *n*= 43, or 21%); boxing had eight females (of *n*= 57, or 14%); and T&K had nine females (of *n*= 29, or 31%).

Step 4. Model 2: Final model – overall as well as by competitive level and primary sport

|  | B | SE | 95% CI | *p* | β | *r* with winning record | *p* | Tolerance | VIF | *M* | *SD* |
| --- | --- | --- | --- | --- | --- | --- | --- | --- | --- | --- | --- |
|  |  |  | Overall |  |  |  |  |  |  |  |  |
| Winning Record |  |  |  |  |  |  |  |  |  | 68.26 | 23.32 |
| *n=* 156; *R*^2^= .37 (*p*< .01) |  |  |  |  |  |  |  |  |  |  |  |
| (Constant) | 83.61 | 6.57 | 70.62; 96.59 | .000 |  |  |  |  |  |  |  |
| Career Stage | .01 | .05 | -.08; .11 | .78 | .02 | -.12 | .06 | .94 | 1.06 | 41.12 | 31.48 |
| Competitive style | .10 | .073 | -.05; .24 | .18 | .09 | .18 | .01 | .95 | 1.05 | 62.73 | 21.29 |
| Losses by points or judge's decision | -6.84 | .91 | -8.63; -5.05 | **<.01** | **-.50** | -.48 | <.01 | .93 | 1.07 | 3.42 | 1.72 |
| Victory by knock-out | 2.75 | 1.35 | .089; 5.41 | **.04** | **.14** | .16 | .02 | .94 | 1.07 | 2.13 | 1.16 |
| Loss by knock-out | -19.58 | 3.67 | -26.83; -12.32 | **<.01** | **-.36** | -.28 | <.01 | .94 | 1.06 | 0.23 | 0.42 |
| *n=* 27; *R*^2^= .48 (*p*< .01) |  |  | MMA |  |  |  |  |  |  |  |  |
| Career Stage | 0.11 | 0.13 | -0.11; 0.33 | .40 | .12 |  |  |  |  |  |  |
| Competitive style | -0.12 | 0.17 | -0.34; .15 | .46 | -.13 |  |  |  |  |  |  |
| Losses by points or judge's decision | -5.62 | 2.06 | -9.01; -2.24 | **<.01** | **-.43** |  |  |  |  |  |  |
| Victory by knock-out | 5.47 | 2.59 | 1.20; 9.73 | **.04** | **.33** |  |  |  |  |  |  |
| Loss by knock-out | -24.32 | 6.77 | -35.45; 13.19 | **<.01** | **-.50** |  |  |  |  |  |  |
| *n=* 43; *R*^2^= .48 (*p*< .01) |  |  | MT / KB |  |  |  |  |  |  |  |  |
| Career Stage | -0.03 | 0.08 | -0.16; 0.10 | .69 | -.04 |  |  |  |  |  |  |
| Competitive style | 0.23 | 0.14 | -0.01; 0.46 | .11 | .18 |  |  |  |  |  |  |
| Losses by points or judge's decision | -8.34 | 1.42 | 10.68; -6.01 | **<.01** | **-.66** |  |  |  |  |  |  |
| Victory by knock-out | 2.00 | 2.23 | -1.77; 5.76 | .38 | .10 |  |  |  |  |  |  |
| Loss by knock-out | -14.42 | 6.04 | -24.35; -4.49 | **.02** | **-.27** |  |  |  |  |  |  |
|  |  |  |  |  |  |  |  |  |  |  |  |
| Table (continued) |  |  |  |  |  |  |  |  |  |  |  |
| *n=* 57; *R*^2^= .44 (*p*< .01) |  |  | Boxing |  |  |  |  |  |  |  |  |
| Career Stage | -0.03 | 0.09 | -0.17; 0.11 | .74 | -.03 |  |  |  |  |  |  |
| Competitive style | 0.19 | 0.13 | -0.03; 0.41 | .16 | .14 |  |  |  |  |  |  |
| Losses by points or judge's decision | -8.55 | 1.62 | -11.21; -5.89 | **<.01** | **-.56** |  |  |  |  |  |  |
| Victory by knock-out | 3.10 | 2.14 | -0.43; 6.62 | .15 | .15 |  |  |  |  |  |  |
| Loss by knock-out | -27.17 | 6.84 | -38.41; -15.92 | **<.01** | **-.41** |  |  |  |  |  |  |
| *n=* 29; *R*^2^= .21 (*p*= .12) |  |  | T&K |  |  |  |  |  |  |  |  |
| Career Stage | 0.06 | 0.12 | -0.13; 0.25 | .60 | .10 |  |  |  |  |  |  |
| Competitive style | 0.19 | 0.16 | -0.08; 0.46 | .24 | .20 |  |  |  |  |  |  |
| Losses by points or judge's decision | -4.74 | 2.56 | -8.95; -0.53 | **.06** | **-0.33** |  |  |  |  |  |  |
| Victory by knock-out | 2.29 | 5.23 | -6.31; 10.88 | .66 | .08 |  |  |  |  |  |  |
| Loss by knock-out | -15.47 | 8.58 | -29.58; -1.36 | **.07** | **-.33** |  |  |  |  |  |  |
| *n=* 84; *R*^2^= .53 (*p*< .01) |  |  | Amateur |  |  |  |  |  |  |  |  |
| Career Stage | -0.01 | 0.07 | -0.12; 0.10 | 0.88 | -.01 |  |  |  |  |  |  |
| Competitive style | 0.13 | 0.10 | -0.03; 0.29 | 0.18 | .11 |  |  |  |  |  |  |
| Losses by points or judge's decision | -8.49 | 1.14 | -10.34; -6.62 | **<.01** | **-.58** |  |  |  |  |  |  |
| Victory by knock-out | 2.30 | 1.66 | -0.43; 5.03 | .20 | .11 |  |  |  |  |  |  |
| Loss by knock-out | -30.04 | 5.27 | -38.71; -21.34 | **<.01** | **-.44** |  |  |  |  |  |  |
| *n=* 29; *R*^2^= .41 (*p*< .01) |  |  | Regional / State |  |  |  |  |  |  |  |  |
| Career Stage | -0.04 | 0.11 | -0.23; 0.15 | .74 | -.05 |  |  |  |  |  |  |
| Competitive style | 0.19 | .14 | -0.04; 0.43 | .18 | .20 |  |  |  |  |  |  |
| Losses by points or judge's decision | -6.78 | 2.26 | -10.50; -3.07 | **<.01** | **-.48** |  |  |  |  |  |  |
| Victory by knock-out | 3.91 | 3.56 | -1.94; 9.76 | .27 | .19 |  |  |  |  |  |  |
| Loss by knock-out | -14.27 | 8.22 | -27.80; -0.74 | .08 | -.27 |  |  |  |  |  |  |
|  |  |  |  |  |  |  |  |  |  |  |  |
| Table (continued) |  |  |  |  |  |  |  |  |  |  |  |
| *n=* 43; *R*^2^= .21 (*p*= .06) |  |  | Elite |  |  |  |  |  |  |  |  |
| Career Stage | 0.13 | 0.08 | 0.00; 0.26 | .10 | .22 |  |  |  |  |  |  |
| Competitive style | -0.13 | 0.13 | -0.33; 0.08 | .32 | -.14 |  |  |  |  |  |  |
| Losses by points or judge's decision | -2.68 | 1.41 | -5.00; -0.37 | .06 | -.27 |  |  |  |  |  |  |
| Victory by knock-out | 4.11 | 2.28 | 0.35; 7.86 | .07 | .26 |  |  |  |  |  |  |
| Loss by knock-out | -7.30 | 5.07 | -15.64; 1.05 | .15 | -.20 |  |  |  |  |  |  |

Note: Values in bold, *p*< .05. VIF= Variance Inflation Factor.**Supplementary file E: Preliminary Analysis for Model 3**

Step 1: Correlation matrix for the initial standard multiple regression analysis (*n*=151).

|  | 1 | 2 | 3 | 4 | 5 | 6 | 7 | 8 | 9 | 10 | 11 | 12 | 13 | 14 | 15 | 16 |
| --- | --- | --- | --- | --- | --- | --- | --- | --- | --- | --- | --- | --- | --- | --- | --- | --- |
| 1. Winning record | 1.00 |  |  |  |  |  |  |  |  |  |  |  |  |  |  |  |
| 2. Age | .04 | 1.00 |  |  |  |  |  |  |  |  |  |  |  |  |  |  |
| 3. Gender | -.09 | -.06 | 1.00 |  |  |  |  |  |  |  |  |  |  |  |  |  |
| 4. BMI | .02 | .37* | .15* | 1.00 |  |  |  |  |  |  |  |  |  |  |  |  |
| 5. Career stage | .21* | .51* | .07 | .43 | 1.00 |  |  |  |  |  |  |  |  |  |  |  |
| 6. Competitive style | .21* | .14* | .00 | -.02 | .06 | 1.00 |  |  |  |  |  |  |  |  |  |  |
| 7. Frequency of *combat* sport training sessions each week *without* a competition in the near-term | -.02 | -.12 | -.10 | -.05 | .03 | .18 | 1.00 |  |  |  |  |  |  |  |  |  |
| 8. Frequency of *non-combat* training sessions each week *without* a competition in the near term | .14* | .05 | .08 | .14 | .20* | .04 | .16* | 1.00 |  |  |  |  |  |  |  |  |
| 9. Frequency of *combat* sport training sessions each week *with* a competition in the near-term | .080 | -.13 | -.06 | -.10 | -.07 | .19* | .68* | .15* | 1.00 |  |  |  |  |  |  |  |
| 10. Frequency of *non-combat* training sessions each week *with* a competition in the near-term | .08 | .07 | .04 | .07 | .16* | .04 | .10 | .83 | .15* | 1.00 |  |  |  |  |  |  |
| 11. Victory by points or judge's decision | -.01 | .07 | .03 | .02 | .22* | -.05 | -.02 | .03 | .02 | .02 | 1.00 |  |  |  |  |  |
| 12. Loss by points or judge's decision | .01 | .07 | -.07 | .08 | .17* | -.04 | .13 | .02 | .02 | .01 | .32* | 1.00 |  |  |  |  |
| 13. Victory by disqualification | .07 | -.07 | .06 | -.02 | .23* | .07 | -.01 | .06 | .01 | .07 | .03 | .01 | 1.00 |  |  |  |
| 14. Loss by disqualification | .07 | .12 | .02 | .11 | .18* | .19* | .04 | .09 | .00 | .02 | -.06 | .11 | .474 | 1.00 |  |  |
| 15. Victory by submission or pin | .05 | .07 | .02 | .05 | .26* | .00 | .18* | .12* | .17* | .07 | -.28* | .03 | .199 | .17 | 1.00 |  |
| 16. Loss by submission or pin | -.27* | -.08 | .15* | -.05 | .03 | -.06 | .06 | .04 | .05 | .00 | .04 | -.30* | .058 | -.05 | .23* | 1.00 |

Note: * *p*< .05

Step 2: Results from initial standard multiple regression analysis (*n*=151).

|  | B | *SE* | 95% CI | *p* | β | *r* with winning record | *p* | Tolerance | VIF | *M* | *SD* |
| --- | --- | --- | --- | --- | --- | --- | --- | --- | --- | --- | --- |
| Winning record |  |  |  |  |  |  |  |  |  | 67.11 | 19.88 |
| (Constant) | 84.39 | 14.67 | 55.39; 113.39 | <.01 |  |  |  |  |  |  |  |
| Age | -.27 | .18 | -.62; .08 | .13 | -.15 | .04 | .30 | .61 | 1.64 | 29.84 | 10.86 |
| Gender | -7.13 | 6.32 | -19.62; 5.36 | .26 | -.09 | -.09 | .15 | .91 | 1.10 | .93 | .25 |
| BMI | -0.30 | 0.40 | -1.08; 0.49 | .46 | -.07 | .02 | .40 | .73 | 1.38 | 25.92 | 4.433 |
| **Career stage** | **0.19** | **0.07** | **0.07; 0.3** | **<.01** | **.32** | **.21** | **<.01** | **.50** | **2.02** | **48.93** | **32.78** |
| **Competitive style** | **0.17** | **0.07** | **0.03; 0.32** | **.02** | **.19** | **.21** | **<.01** | **.88** | **1.14** | **63.94** | **21.63** |
| Frequency of *combat* sport training sessions each week *without* a competition in the near-term | -2.01 | 1.08 | -4.14; 0.12 | .06 | -.20 | -.02 | .40 | .51 | 1.95 | 4.35 | 1.95 |
| Frequency of *non-combat* training sessions each week *without* a competition in the near term | 2.38 | 1.35 | -0.29; 5.04 | .08 | .25 | .14 | .05 | .29 | 3.46 | 3.21 | 2.08 |
| Frequency of *combat* sport training sessions each week *with* a competition in the near-term | 1.33 | 0.85 | -0.35; .01 | .12 | .16 | .08 | .16 | .52 | 1.92 | 5.62 | 2.45 |
| Frequency of *non-combat* training sessions each week *with* a competition in the near-term | -1.59 | 1.28 | -4.13; 0.95 | .22 | -.17 | .08 | .16 | .30 | 3.35 | 3.30 | 2.14 |
| Victory by points or judge's decision | -0.11 | 1.51 | -3.09; 2.88 | .95 | -.01 | -.01 | .46 | .67 | 1.49 | 2.83 | 1.21 |
| Loss by points or judge's decision | -1.60 | 1.32 | -4.21; 1.02 | .23 | -.11 | .01 | .46 | .72 | 1.39 | 2.84 | 1.34 |
| Victory by disqualification | -0.31 | 4.65 | -9.45; 8.88 | .95 | -.01 | .07 | .18 | .67 | 1.50 | .19 | .40 |
| Loss by disqualification | -0.83 | 5.43 | -11.56; 9.91 | .88 | -.01 | .07 | .19 | .69 | 1.45 | .13 | .33 |
| Victory by submission or pin | .92 | 1.86 | -2.75; 4.59 | .62 | .05 | .05 | .29 | .67 | 1.50 | 2.87 | 1.00 |
| **Loss by submission or pin** | **-6.19** | **1.66** | **-9.48; -2.89** | **<.01** | **-.32** | **-.27** | **<.01** | **.77** | **1.30** | **2.29** | **1.03** |

Note: Values in bold, *p*< .05. VIF= Variance Inflation Factor.

**Observations and decisions**

The following variables were retained as they were statistically significant contributors to explaining variance in winning record: career stage, competitive style, and loss by submission or pin.

Step 3: Moderation analyses.

|  | Competitive Level (*df*= 2) | Primary Sport (*df*= 3) |
| --- | --- | --- |
| Age | χ^2^= 1.96, *p*= .37 | χ^2^= 1.65, *p*= .65 |
| Gender | χ^2^= 1.41, *p*= .49 | χ^2^= 2.90, *p*= .41 |
| BMI | χ^2^= 0.12, *p*= .94 | χ^2^= 1.06, *p*= .79 |
| Career stage | χ^2^= 0.11, *p*= .95 | χ^2^= 2.24, *p*= .53 |
| Competitive style | χ^2^= 0.67, *p*= .72 | χ^2^= 0.49, *p*= .92 |
| Frequency of *combat* sport training sessions each week *without* a competition in the near-term | χ^2^= 5.11, *p*= .08 | χ^2^= 5.34, *p*= .15 |
| **Frequency of *non-combat* training sessions each week *without* a competition in the near term** | **χ^2^= 6.52, *p*= .04** | **χ^2^= 23.96, *p*< .01** |
| Frequency of *combat* sport training sessions each week *with* a competition in the near-term | χ^2^= 4.17, *p*= .12 | χ^2^= 1.42, *p*= .70 |
| **Frequency of *non-combat* training sessions each week *with* a competition in the near-term** | **χ^2^= 12.79, *p*<.01** | **χ^2^= 16.04, *p*< .01** |
| Victory by points or judge's decision | χ^2^= 3.31, *p*= .19 | χ^2^= 0.59, *p*= .90 |
| Loss by points or judge's decision | χ^2^= 1.21, *p*= .55 | **χ^2^= 17.00, *p*< .01** |
| Victory by disqualification | χ^2^= 0.03, *p*= .99 | χ^2^= 2.61, *p*= .46 |
| Loss by disqualification | χ^2^= 0.09, *p*= .96 | χ^2^= 2.96, *p*= .34 |
| Victory by submission or pin | χ^2^= 0.49, *p*= .78 | χ^2^= 6.07, *p*= .11 |
| Loss by submission or pin | χ^2^= 2.34, *p*= .31 | χ^2^= 4.58, *p*= .21 |

Note: Values in bold, *p*< .05.

**Observations and decisions**

As moderation effects were observed for frequency of *non-combat* training sessions each week *without* a competition in the near term, frequency of *non-combat* training sessions each week *with* a competition in the near-term, and loss by points or judge's decision, it was decided to retain these variables for the final model.

Step 4. Model 3: Final model – overall as well as by competitive level and primary sport

|  | B | *SE* | 95% CI | *p*. | β | *r* with winning record | p | Tolerance | VIF | M | SD |
| --- | --- | --- | --- | --- | --- | --- | --- | --- | --- | --- | --- |
|  |  |  | Overall |  |  |  |  |  |  |  |  |
| Winning record |  |  |  |  |  |  |  |  |  | 67.11 | 19.88 |
| *n=* 151; *R*^2^ = .18, *p*< .01 |  |  |  |  |  |  |  |  |  |  |  |
| (Constant) | 67.37 | 7.85 | 51.85; 2.90 | <.01 |  |  |  |  |  |  |  |
| Career stage | .123 | .05 | .03; .22 | **.01** | **.20** | .21 | <.01 | .92 | 1.08 | 48.93 | 32.78 |
| Competitive style | .16 | .07 | .02; .30 | **.03** | **.17** | .21 | <.01 | .99 | 1.02 | 63.94 | 21.63 |
| Frequency of *non-combat* training sessions each week *without* a competition in the near term | 2.03 | 1.30 | -.54; 4.60 | .12 | .21 | .14 | .05 | .31 | 3.24 | 3.21 | 2.08 |
| Frequency of *non-combat* training sessions each week *with* a competition in the near term | -1.24 | 1.25 | -3.71; 1.24 | .33 | -.13 | .08 | .16 | .31 | 3.19 | 3.30 | 2.14 |
| Loss by submission or pin | -6.02 | 1.54 | -9.07; -2.97 | **<.01** | **-.31** | -.27 | <.01 | .89 | 1.12 | 2.84 | 1.34 |
| Loss by points or judge's decision | -1.75 | 1.20 | -4.11; 0.622 | .15 | -.12 | .01 | .46 | .87 | 1.15 | 2.29 | 1.03 |
| *n=* 27; *R*^2^ = .21, *p*= .13 |  |  | MMA |  |  |  |  |  |  |  |  |
| Career stage | 0.07 | 0.18 | -0.22 0.37 | .69 | .08 |  |  |  |  |  |  |
| Competitive style | -0.06 | 0.22 | -0.41; .30 | .80 | -.06 |  |  |  |  |  |  |
| Frequency of *non-combat* training sessions each week *without* a competition in the near term | -3.55 | 3.72 | -9.66; 2.57 | .34 | -.40 |  |  |  |  |  |  |
| Frequency of *non-combat* training sessions each week *with* a competition in the near term | 3.83 | 3.96 | -2.68; 10.34 | .33 | .43 |  |  |  |  |  |  |
| Loss by submission or pin | -1.06 | 3.70 | -7.14; 5.02 | .77 | -.06 |  |  |  |  |  |  |
| Loss by points or judge's decision | -5.33 | 2.66 | -9.71; -0.96 | .05 | -0.41 |  |  |  |  |  |  |
|  |  |  |  |  |  |  |  |  |  |  |  |
|  |  |  |  |  |  |  |  |  |  |  |  |
|  |  |  |  |  |  |  |  |  |  |  |  |
|  |  |  |  |  |  |  |  |  |  |  |  |
|  |  |  |  |  |  |  |  |  |  |  |  |
|  |  |  |  |  |  |  |  |  |  |  |  |
|  |  |  |  |  |  |  |  |  |  |  |  |
| Table (continued) |  |  |  |  |  |  |  |  |  |  |  |
| *n=* 56; *R*^2^ = .39, *p*< .01 |  |  | BJJ |  |  |  |  |  |  |  |  |
| Career stage | 0.17 | 0.08 | 0.04; 0.29 | **.03** | **.23** |  |  |  |  |  |  |
| Competitive style | 0.19 | 0.11 | 0.01; 0.37 | .08 | .19 |  |  |  |  |  |  |
| Frequency of *non-combat* training sessions each week *without* a competition in the near term | 6.89 | 2.34 | 3.04; 10.74 | **<.01** | **.46** |  |  |  |  |  |  |
| Frequency of *non-combat* training sessions each week *with* a competition in the near term | -1.64 | 1.95 | -4.85; 1.57 | .40 | -.12 |  |  |  |  |  |  |
| Loss by submission or pin | -5.59 | 2.58 | 9.84; -1.35 | **.03** | **-.29** |  |  |  |  |  |  |
| Loss by points or judge's decision | -1.80 | 2.18 | -5.38; 1.78 | .41 | -.11 |  |  |  |  |  |  |
| *n=* 42; *R*^2^ = .42, *p*< .01 |  |  | Wrestling |  |  |  |  |  |  |  |  |
| Career stage | -0.03 | 0.06 | -0.13; 0.08 | .68 | -.05 |  |  |  |  |  |  |
| Competitive style | 0.03 | 0.09 | 0.11; 0.17 | .74 | .04 |  |  |  |  |  |  |
| Frequency of *non-combat* training sessions each week *without* a competition in the near term | 0.18 | 1.40 | -2.12; 2.48 | .90 | .03 |  |  |  |  |  |  |
| Frequency of *non-combat* training sessions each week *with* a competition in the near term | -1.93 | 1.29 | -4.05; 0.20 | .14 | -.30 |  |  |  |  |  |  |
| Loss by submission or pin | -6.07 | 2.49 | -10.17; -1.96 | **.02** | **-.32** |  |  |  |  |  |  |
| Losse by points or judge's decision | 4.94 | 2.15 | 1.41; 8.45 | **.02** | **.30** |  |  |  |  |  |  |
| *n=* 26; *R*^2^ = .25, *p*< .09 |  |  | Judo |  |  |  |  |  |  |  |  |
| Career stage | 0.12 | 0.14 | -0.12; 0.35 | .41 | .17 |  |  |  |  |  |  |
| Competitive style | 0.13 | 0.21 | -0.22; 0.48 | .54 | .13 |  |  |  |  |  |  |
| Frequency of *non-combat* training sessions each week *without* a competition in the near term | 4.87 | 4.16 | -1.97; 11.71 | .24 | .40 |  |  |  |  |  |  |
| Frequency of *non-combat* training sessions each week *with* a competition in the near term | -0.42 | 4.05 | -7.08; 6.25 | .92 | -.03 |  |  |  |  |  |  |
| Loss by submission or pin | -5.06 | 4.09 | 11.78; 1.66 | .22 | -.22 |  |  |  |  |  |  |
| Loss by points or judge's decision | -1.92 | 3.50 | -7.7; 3.84 | .58 | -.11 |  |  |  |  |  |  |
|  |  |  |  |  |  |  |  |  |  |  |  |
|  |  |  |  |  |  |  |  |  |  |  |  |
|  |  |  |  |  |  |  |  |  |  |  |  |
|  |  |  |  |  |  |  |  |  |  |  |  |
|  |  |  |  |  |  |  |  |  |  |  |  |
| Table (continued) |  |  |  |  |  |  |  |  |  |  |  |
| *n=* 37; *R*^2^ = .29, *p*= .02 |  |  | Amateurs |  |  |  |  |  |  |  |  |
| Career stage | 0.27 | 0.11 | 0.09; .44 | **.01** | **.43** |  |  |  |  |  |  |
| Competitive style | 0.05 | 0.15 | -0.29; 0.19 | .74 | -.06 |  |  |  |  |  |  |
| Frequency of *non-combat* training sessions each week *without* a competition in the near term | 3.56 | 2.51 | -0.57; 7.69 | .16 | .38 |  |  |  |  |  |  |
| Frequency of *non-combat* training sessions each week *with* a competition in the near term | -2.75 | 2.345 | -6.62; 1.11 | .241.241 | -.33 |  |  |  |  |  |  |
| Loss by submission or pin | -9.26 | 3.62 | -15.22; -3.31 | **.01** | **-.48** |  |  |  |  |  |  |
| Loss by points or judge's decision | -6.06 | 2.47 | -10.13;-1.99 | **.01** | **-.41** |  |  |  |  |  |  |
| *n=* 69; *R*^2^ = .36, *p*< .01 |  |  | Regional / State |  |  |  |  |  |  |  |  |
| Career stage | 0.08 | 0.07 | -0.03; 0.19 | .21 | .13 |  |  |  |  |  |  |
| Competitive style | 0.28 | 0.10 | 0.12; 0.43 | **<.01** | **.28** |  |  |  |  |  |  |
| Frequency of *non-combat* training sessions each week *without* a competition in the near term | 1.59 | 1.83 | -1.42; 4.60 | .38 | .13 |  |  |  |  |  |  |
| Frequency of *non-combat* training sessions each week *with* a competition in the near term | 2.60 | 1.73 | -0.24; 5.45 | .13 | .23 |  |  |  |  |  |  |
| Loss by submission or pin | -8.09 | 2.08 | -11.51; -4.67 | **<.01** | **-.41** |  |  |  |  |  |  |
| Loss by points or judge's decision | -1.94 | 1.65 | -5.17; -1.94 | .24 | -.12 |  |  |  |  |  |  |
| *n=* 45; *R*^2^ = .16, *p*= .12 |  |  | Elite |  |  |  |  |  |  |  |  |
| Career stage | 0.08 | 0.08 | -0.06; 0.21 | .34 | .14 |  |  |  |  |  |  |
| Competitive style | 0.09 | 0.12 | -0.11; 0.29 | .48 | .10 |  |  |  |  |  |  |
| Frequency of *non-combat* training sessions each week *without* a competition in the near term | 2.69 | 2.08 | -0.73; 6.10 | .20 | .36 |  |  |  |  |  |  |
| Frequency of *non-combat* training sessions each week *with* a competition in the near term | -4.50 | 2.17 | -8.06; -0.93 | **.04** | **-.58** |  |  |  |  |  |  |
| Loss by submission or pin | -3.06 | 2.64 | -7.41; 1.29 | .25 | -.17 |  |  |  |  |  |  |
| Loss by points or judge's decision | -1.19 | 1.90 | -4.31; 1.93 | .53 | -.09 |  |  |  |  |  |  |

Note: Values in bold, *p*< .05. VIF= Variance Inflation Factor.

**Supplementary file F: Preliminary Analysis for Model 4**

Step 1: Correlation matrix for the initial standard multiple regression analysis (*n*=68).

|  | 1 | 2 | 3 | 4 | 5 | 6 | 7 | 8 | 9 | 10 | 11 | 12 | 13 | 14 | 15 | 16 | 17 | 18 |
| --- | --- | --- | --- | --- | --- | --- | --- | --- | --- | --- | --- | --- | --- | --- | --- | --- | --- | --- |
| 1. Winning record | 1.00 |  |  |  |  |  |  |  |  |  |  |  |  |  |  |  |  |  |
| 2. Age | .05 | 1.00 |  |  |  |  |  |  |  |  |  |  |  |  |  |  |  |  |
| 3. Gender | -.19 | .16 | 1.00 |  |  |  |  |  |  |  |  |  |  |  |  |  |  |  |
| 4. BMI | .01 | .47* | .08 | 1.00 |  |  |  |  |  |  |  |  |  |  |  |  |  |  |
| 5. Career stage | .22* | .53* | .08 | .46* | 1.00 |  |  |  |  |  |  |  |  |  |  |  |  |  |
| 6. Competitive style | .22* | .22* | .16 | .11 | .17* | 1.00 |  |  |  |  |  |  |  |  |  |  |  |  |
| Frequency of *combat* sport training sessions each week *without* a competition in the near-term | .01 | -.10 | -.04 | -.04 | .16* | .26 | 1.00 |  |  |  |  |  |  |  |  |  |  |  |
| Frequency of *non-combat* training sessions each week *without* a competition in the near term | .04 | .05 | -.01 | -.05 | .09 | .04 | .34* | 1.00 |  |  |  |  |  |  |  |  |  |  |
| Frequency of *combat* sport training sessions each week *with* a competition in the near-term | .07 | -.18 | .02 | -.14 | -.08 | .28* | .48* | .27* | 1.00 |  |  |  |  |  |  |  |  |  |
| Frequency of *non-combat* training sessions each week *with* a competition in the near-term | -.03 | .06 | -.02 | -.05 | .06 | -.07 | .16 | .83* | .17 | 1.00 |  |  |  |  |  |  |  |  |
| 11. Victories Points or judge's decision | .17 | .08 | .21* | .01 | .30* | .13 | .11 | .18* | .22 | .17* | 1.00 |  |  |  |  |  |  |  |
| 12. Losses Points or judge's decision | .38* | .04 | .05 | .12 | .40* | .25* | .34* | .11 | .18 | .05 | .61* | 1.00 |  |  |  |  |  |  |
| 13. Victories Disqualification | .02 | -.11 | .11 | .01 | .17* | .08 | -.13 | -.13 | -.04 | -.07 | -.06 | .06 | 1.00 |  |  |  |  |  |
| 14. Losses Disqualification | .00 | .07 | .09 | .17 | .12 | .16* | .14* | -.04 | .07 | -.13 | -.12 | .06 | .51* | 1.00 |  |  |  |  |
| 15. Victories Submission or pin | .03 | -.08 | -.12 | .05 | .22* | -.01 | .26* | .07 | .17 | .05 | -.25* | .13 | .18* | .18* | 1.00 |  |  |  |
| 16. Losses Submission or pin | -.33* | -.15 | .18* | -.06 | -.07 | -.11 | -.08 | .12 | .01 | .09 | .04 | -.19* | -.09 | -.03 | .00 | 1.00 |  |  |
| 17. Victories Ippon, Waza-ari or technical fall | .33* | -.04 | .14 | .05 | .17* | .09 | -.18* | -.26* | -.06 | -.28* | -.18 | .09 | .31* | .24* | .05 | -.10 | 1.00 |  |
| 18. Losses Ippon or Waza-ari, technical fall | -.14 | -.09 | -.13 | .00 | .07 | -.22* | -.15* | -.25* | -.16 | -.20* | -.26* | -.05 | .29* | .06 | .04 | -.06 | .48* | 1.00 |

Note: * *p*< .05

Step 2: Results from initial standard multiple regression analysis (*n*= 68).

| *R*^2^= .51, *p*< .01 | B | SE | 95 % CI | *p*. | β | *r* with winning record | *p* | Tolerance | VIF | *M* | *SD* |
| --- | --- | --- | --- | --- | --- | --- | --- | --- | --- | --- | --- |
| Winning record |  |  |  |  |  |  |  |  |  | 67.82 | 18.62 |
| (Constant) | 96.49 | 20.95 | 54.43; 138.56 | <.01 |  |  |  |  |  |  |  |
| Age | .00 | .22 | -.45; .45 | 1.00 | .00 | .05 | .33 | .43 | 2.35 | 31.24 | 12.62 |
| Gender | -33.85 | 12.38 | -58.72; -8.98 | **<.01** | -.31 | -.19 | .06 | .76 | 1.31 | .97 | .170 |
| BMI | -.27 | .45 | -1.19; 0.64 | .55 | -.07 | .01 | .46 | .65 | 1.53 | 26.69 | 5.01 |
| Career stage | .07 | .10 | -.13; .26 | .50 | .12 | .22 | .04 | .34 | 2.93 | 64.31 | 32.62 |
| Competitive style | .05 | .12 | -.16; .25 | .66 | .05 | .22 | .04 | .67 | 1.50 | 62.50 | 22.05 |
| Frequency of *combat* sport training sessions each week *without* a competition in the near-term | -1.40 | 1.40 | -4.22; 1.42 | .32 | -.14 | .10 | .47 | .47 | 2.13 | 4.07 | 1.91 |
| Frequency of *non-combat* training sessions each week *without* a competition in the near term | 1.92 | 1.65 | -1.39; 5.23 | .25 | .24 | .04 | .36 | .24 | 4.14 | 3.85 | 2.27 |
| Frequency of *combat* sport training sessions each week *with* a competition in the near-term | 0.18 | 0.92 | -1.66; 2.02 | .85 | .03 | .07 | .28 | .59 | 1.70 | 5.32 | 2.62 |
| Frequency of *non-combat* training sessions each week *with* a competition in the near-term | -1.28 | 1.51 | -4.32; 1.76 | .40 | -.16 | -.03 | .39 | .26 | 3.86 | 3.79 | 2.39 |
| Victory by points or judge's decision | -.05 | 2.89 | -5.86; 5.76 | .99 | .00 | .17 | .08 | .31 | 3.25 | 2.90 | 1.15 |
| Loss by points or judge's decision | 3.99 | 2.39 | -0.82; 8.79 | .10 | .27 | **.38** | **<.01** | .38 | 2.65 | 2.84 | 1.25 |
| Victory by disqualification | 0.73 | 5.76 | -10.83; 12.29 | .90 | .02 | .02 | .43 | .49 | 2.06 | .29 | .46 |
| Loss by disqualification | -4.82 | 6.18 | -17.22; 7.59 | .44 | -.10 | .00 | .50 | .57 | 1.77 | .19 | .40 |
| Victory by submission or pin | -0.87 | 2.91 | -6.72; 4.98 | .77 | -.04 | .03 | .42 | .60 | 1.67 | 2.93 | .82 |
| Loss by submission or pin | -4.65 | 2.41 | -9.49; 0.20 | .06 | -.22 | **-.33** | **<.01** | .78 | 1.28 | 2.37 | .86 |
| **Victory by Ippon, Waza-ari or technical fall** | **24.46** | **6.46** | **11.49; 37.42** | **<.01** | **.52** | **.33** | **<.01** | **.52** | **1.93** | **.81** | **.40** |
| **Loss by Ippon or Waza-ari, technical fall** | **-15.92** | **4.94** | **-25.85; -5.99** | **<.01** | **-.42** | **-.14** | **.13** | **.59** | **1.70** | **.63** | **.49** |

Note: Values in bold, *p*< .05. VIF= Variance Inflation Factor.

**Observations and decisions**:

Victory and loss by Ippon, Waza-ari or technical fall were retained as these variables were retained were statistically significant contributors to explaining variance in winning record and the correlation between the two variables was not large but not excessively so (*r*= .48).

Loss by points or judge's decision and loss by submission or pin were also retained because the Pearson *r* between these variables and winning record were positive and statistically significant but these effects were suppressed in the initial multiple regression analysis. The likely source of statistical suppression for loss by points or judge's decision was the large and significant positive correlation (*r*= .62) with victories by points or judge's decision for this sample (that is, wrestling and judo combat sport athletes). There was no obvious source for the statistical suppression for loss by submission or pin.

Gender was not retained for the final model as there were only two females in the sample of *n*= 68.l.

Step 3: Moderation analyses.

|  | Competitive Level (*df*= 2) | Primary Sport (*df*= 1) |
| --- | --- | --- |
| Age | Not reported as *n*=9 for Amateurs | χ^2^= 1.26, *p*= .26 |
| BMI |  | χ^2^= 0.78, *p*= .38 |
| Career stage |  | χ^2^= 0.15, *p*= .69 |
| Competitive style |  | χ^2^= 0.44, *p*= .51 |
| Frequency of *combat* sport training sessions each week *without* a competition in the near-term |  | χ^2^= 2.64, *p*= .10 |
| **Frequency of *non-combat* training sessions each week *without* a competition in the near term** |  | **χ^2^= 8.68, *p*<.01** |
| Frequency of *combat* sport training sessions each week *with* a competition in the near-term |  | χ^2^= 0.76, *p*= .38 |
| **Frequency of *non-combat* training sessions each week *with* a competition in the near-term** |  | **χ^2^= 7.80, *p*<.01** |
| Victory by points or judge's decision |  | χ^2^= 0.14, *p*= .71 |
| Loss by points or judge's decision |  | χ^2^= 2.33, *p*= .13 |
| Victory by disqualification |  | χ^2^= 0.86, *p*= .35 |
| Loss by disqualification |  | χ^2^= 0.00, *p*= .99 |
| Victory by submission or pin |  | χ^2^= 1.99, *p*= .16 |
| Loss by submission or pin |  | χ^2^= 0.40, *p*= .53 |
| Victory by Ippon, Waza-ari or technical fall |  | χ^2^= 0.26, *p*= .61 |
| **Loss by Ippon or Waza-ari, technical fall** |  | **χ^2^= 10.13, *p*<.01** |

**Observations and decisions**

With both *non-combat* training variables showing similar moderation effects, both variables were options to be retained for the final model. It was decided to retain the variable relating to competition in the near term as such a variable would be more salient implications for athletes and coaches. Repeating this model with only one of the variables in the model did not provide any evidence as to the superiority of the one variable over the other as the results were almost identical.

Step 4. Model 4: Final model – overall as well as by primary sport

|  | B | *SE* | 95% CI | *p* | β | *r* with Winning Record | *p* | Tolerance | VIF | *M* | *SD* |
| --- | --- | --- | --- | --- | --- | --- | --- | --- | --- | --- | --- |
| Overall | | | | | | | | | | | |
| Winning Record |  |  |  |  |  |  |  |  |  | 67.82 | 18.62 |
| *n*= 68; *R^2^*= 39, *p*< .01 |  |  |  |  |  |  |  |  |  |  |  |
| (Constant) | 59.70 | 9.28 | 41.14; 78.25 | <.01 |  |  |  |  |  |  |  |
| Frequency of *non-combat* training sessions each week *with* a competition in the near-term | 0.26 | 0.81 | -1.36; 1.89 | .75 | .03 | -.03 | .39 | .91 | 1.10 | 3.79 | 2.39 |
| Victory by Ippon, Waza-ari or technical fall | 21.92 | 5.52 | 10.88; 32.95 | **<.01** | **.47** | .33 | <.01 | .72 | 1.40 | .81 | .40 |
| Loss by Ippon or Waza-ari, technical fall | -13.8 | 4.39 | -22.57; -5.03 | **<.01** | **-.36** | -.19 | .13 | .76 | 1.33 | .63 | .49 |
| Loss by points or judge's decision | 3.96 | 1.52 | 0.91; 7.01 | **.01** | **.27** | .38 | <.01 | .94 | 1.07 | 2.84 | 1.25 |
| Loss by submission or pin | -5.54 | 2.20 | -9.94; -1.13 | **.02** | **-.26** | -.33 | <.01 | .95 | 1.05 | 2.37 | .86 |
| Wrestling | | | | | | | | | | | |
| *n*= 42; *R^2^*= 52, *p*< .01 |  |  |  |  |  |  |  |  |  |  |  |
| Frequency of *non-combat* training sessions each week *with* a competition in the near-term | -1.45 | .72 | -2.63; -0.26 | **.05** | **-.23** |  |  |  |  |  |  |
| Victory by Ippon, Waza-ari or technical fall | 14.78 | 4.85 | 6.80; 22.76 | **<.01** | **.41** |  |  |  |  |  |  |
| Loss by Ippon or Waza-ari, technical fall | -3.51 | 3.97 | -10.04; 3.03 | .38 | -.11 |  |  |  |  |  |  |
| Loss by points or judge's decision | 2.81 | 2.15 | -0.73; 6.35 | .19 | .17 |  |  |  |  |  |  |
| Loss by submission or pin | -5.06 | 2.24 | -8.74; -1.39 | **.02** | **-.26** |  |  |  |  |  |  |
| Judo | | | | | | | | | | | |
| *n= 26; R^2^*= 59, *p*< .01 |  |  |  |  |  |  |  |  |  |  |  |
| Frequency of *non-combat* training sessions each week *with* a competition in the near-term | 4.05 | 1.58 | 1.45; 6.65 | **.01** | **.33** |  |  |  |  |  |  |
| Victory by Ippon, Waza-ari or technical fall | 34.20 | 11.23 | 15.72; 52.68 | **<.01** | **.44** |  |  |  |  |  |  |
| Loss by Ippon or Waza-ari, technical fall | -36.28 | 7.71 | -48.96; 23.61 | **<.01** | **-.63** |  |  |  |  |  |  |
| Loss by points or judge's decision | -0.49 | 2.25 | -4.19; 3.22 | .83 | -.03 |  |  |  |  |  |  |
| Loss by submission or pin | -6.79 | 3.26 | -12.15; -1.42 | **.04** | **-.29** |  |  |  |  |  |  |

Note: Values in bold, *p*< .05. VIF= Variance Inflation Factor.
